# Effects of Chronic Stress on Prefrontal Cortex Transcriptome in Mice Displaying Different Genetic Backgrounds

**DOI:** 10.1007/s12031-012-9850-1

**Published:** 2012-07-27

**Authors:** Pawel Lisowski, Marek Wieczorek, Joanna Goscik, Grzegorz R. Juszczak, Adrian M. Stankiewicz, Lech Zwierzchowski, Artur H. Swiergiel

**Affiliations:** 1 Department of Molecular Biology, Institute of Genetics and Animal Breeding, Polish Academy of Sciences, Postepu 1, 05-552 Jastrzebiec n/Warsaw, Warsaw, Poland; 2 Department of Neurobiology, Faculty of Biology and Environmental Protection, University of Lodz, Pomorska 141/143, 90-236 Lodz, Poland; 3 Centre for Experimental Medicine, Medical University of Bialystok, Marii Sklodowskiej-Curie 24A, 15-276 Bialystok, Poland; 4 Department of Software Engineering, Bialystok Technical University, Wiejska 45A, 15-351 Bialystok, Poland; 5 Department of Animal Behavior, Institute of Genetics and Animal Breeding, Polish Academy of Sciences, Postepu 1, 05-552 Jastrzebiec n/Warsaw, Warsaw, Poland; 6 Department of Animal Physiology, Institute of Biology, Gdansk University, Kladki 4, 80-882 Gdansk, Poland

**Keywords:** Analgesia, Chronic mild stress, Gene expression, Microarray, Mouse, Pain, Prefrontal cortex, Transcriptome, Transthyretin

## Abstract

There is increasing evidence that depression derives from the impact of environmental pressure on genetically susceptible individuals. We analyzed the effects of chronic mild stress (CMS) on prefrontal cortex transcriptome of two strains of mice bred for high (HA)and low (LA) swim stress-induced analgesia that differ in basal transcriptomic profiles and depression-like behaviors. We found that CMS affected 96 and 92 genes in HA and LA mice, respectively. Among genes with the same expression pattern in both strains after CMS, we observed robust upregulation of *Ttr* gene coding transthyretin involved in amyloidosis, seizures, stroke-like episodes, or dementia. Strain-specific HA transcriptome affected by CMS was associated with deregulation of genes involved in insulin secretion (*Acvr1c*, *Nnat*, and *Pfkm*), neuropeptide hormone activity (*Nts* and *Trh*), and dopamine receptor mediated signaling pathway (*Clic6*, *Drd1a*, and *Ppp1r1b*). LA transcriptome affected by CMS was associated with genes involved in behavioral response to stimulus (*Fcer1g*, *Rasd2*, *S100a8*, *S100a9*, *Crhr1*, *Grm5*, and *Prkcc*), immune effector processes (*Fcer1g*, *Mpo*, and *Igh-VJ558*), diacylglycerol binding (*Rasgrp1*, *Dgke*, *Dgkg*, and *Prkcc*), and long-term depression (*Crhr1*, *Grm5*, and *Prkcc*) and/or coding elements of dendrites (*Crmp1*, *Cntnap4*, and *Prkcc*) and myelin proteins (*Gpm6a*, *Mal*, and *Mog*). The results indicate significant contribution of genetic background to differences in stress response gene expression in the mouse prefrontal cortex.

**Electronic supplementary material:**

The online version of this article (doi:10.1007/s12031-012-9850-1) contains supplementary material, which is available to authorized users.

## Introduction

Exposure to stress is thought to precipitate several neuropsychiatric disorders (Mazure et al*.* 1995). Chronic stress has significant impact on the cellular integrity and function of certain brain areas, most notably the limbic structures (Joels et al*.* 2007; McEwen 2006). In most studies, the hippocampal formation has been investigated as a crucial structure, but recently the prefrontal cortex (PFC) has been seen as equally important (Czeh et al*.* 2008).

Prefrontal cortex in rodents participates in the higher-order functions including learning, memory, event association, the temporal sequencing of tasks, specific aspects of locomotor activity, spatial navigation, decision making, and goal-directed behavior (Pirot et al. 1994; Vertes 2006). PFC plays a key role in working memory, recalling memories from long-term storage, as well as recent memories to guide behavior, while inhibiting inappropriate responses and distractions (Ramos and Arnsten 2007; Robbins 1996). All of these abilities depend on proper PFC network connections, which are vulnerable to stress and neurochemical environment (Arnsten 2009). PFC contributes to negative feedback control of the hypothalamic–pituitary–adrenal axis (HPA) (Herman et al*.* 2003) and regulates the stress responses of other structures (Amat et al*.* 2005; Pascucci et al. 2007). Based on observations from clinical, neuropsychological, and neuroimaging studies, dysfunction of the PFC has been suspected to be accountable for some depressive symptoms (Cummings 1992; Deutch 1993; Fibiger 1995). Dolan et al. (1994) have provided evidence that neuropsychological symptoms in depression are associated with profound hypometabolism, particularly involving the medial PFC (Dolan et al. 1994). Both bipolar and unipolar affective disorders can be identified by decreases in cerebral blood flow and the rate of glucose metabolism in the PFC (Drevets 2000; Drevets et al*.* 1997).

Animal studies indicate that exposure to acute or chronic stress can alter the activity of the neuroendocrine and neurotransmitter systems that affect behavior. Stress in rodents induces anxiety, enhanced fear, anhedonia, and depression (Bekris et al. 2005; Bergstrom et al. 2008; D’Aquila et al. 1994; Wood et al*.* 2008). Chronic stress reduces dopaminergic and serotonergic transmission in the PFC (Mizoguchi et al*.* 2002) and results in a depressive state. Exposure to mild uncontrollable stress impairs PFC functions in humans and animals (Arnsten 2009). Loss of self-control during stress can lead to maladaptive behaviors such as alcohol and drug addiction, smoking, and overeating (Li and Sinha 2008). Stress can also exacerbate the symptoms of bipolar disorders and schizophrenia (Breier et al*.* 1991; Dohrenwend 1994).

Preclinical observations can help to understand stress-related processes in the human brain of genetically stress-vulnerable individuals. Because the responses should differ between the subjects displaying different sensitivity to stress, in the present study we investigated the effects of chronic mild stress (CMS) on gene expression in the frontal cortex of mice selected for high (HA strain) or low (LA strain) stress reaction measured by magnitude of swim stress-induced analgesia (Panocka et al*.* 1986b). The unstressed strains display profound differences in a number of behavioral tests reflecting anxiety or depression. HA mice exhibit higher acoustic startle response (Błaszczyk et al. 2000; Juszczak et al. 2008a) and longer depression-like behavior (immobility) in the tail suspension (TST) and forced swim (FST) tests (Juszczak et al*.* 2008b, 2006; Panocka et al*.* 2001) than the LA strain. The strains also differ in responses to a variety of antidepressants. Desipramine (a prototypic tricylic antidepressant), venlafaxine (selective serotonin reuptake inhibitor), and aminosenktide (tachykinin NK3—receptor agonist) shortened the immobility time of HA mice in the FST or TST, but were ineffective in the LA strain (Juszczak et al. 2006; Panocka et al*.* 2001).

Using gene expression profiling and bioinformatics methodology we now attempted to identify candidate genes, physiological pathways, and potential mechanisms of mood disorders in the PFC of mice that differ in depression-like responses and are exposed to CMS.

## Experimental Procedures

### Animals

Male Swiss Webster mice (weighing 25–30 g, 12 weeks of age), from two lines selected for 76 generations for high (HA) and low (LA) swim stress-induced analgesia (SIA) were used (Panocka et al. 1986a). Adult males and females from each generation, after completion of 3-min swim in 20 °C water, were tested for pain sensitivity on a hot-plate heated to 56 °C. Latency of characteristic hind paw flick or lick response was scored. The animals displaying the longest (50–60 s) and the shortest (<10 s) post-swim latencies of the nociceptive response were chosen for further breeding. Animals were given ordinary daily care with free access to food and water and kept at ambient temperature of 22 ± 1 °C. All procedures had been approved by the Local Ethics Commission and carried out in accordance with the Guiding Principles for the Care and Use of Research Animals.

### Chronic Mild Stress

The animals were assigned to two treatment groups. Control animals (control HA mice, *n* = 15, and control LA mice, *n* = 15) were given ordinary daily care with free access to food and water, while the stressed animals were exposed for 5 weeks to chronic mild stress (CMS HA mice, *n* = 15, and CMS LA mice, *n* = 15). CMS was adapted from the procedures developed by Willner et al. (1987) and used in our previous study, Lisowski et al. (2011). Stressors were applied in a pseudo-random manner during both light and dark phases. All mice received the same treatment schedule, with treatments occurring in different orders in different weeks. The control and CMS groups of mice were housed in single cages and separately in different rooms.

### Sample Preparation

Two days after the end of the CMS, the animals were gently removed from their cages and quickly decapitated within 30 s, the brains were removed and placed on ice-cold glass dish, and prefrontal cortices were immediately isolated (Hamon 2006), aliquoted into freezing vials, frozen in liquid nitrogen, and stored at −80 °C until analysis. Total RNA was isolated, separately from each prefrontal cortex, using NucleoSpin RNA II kit (Macherey-Nagel, Germany), according to the manufacturer's protocol. Nanodrop (Nanodrop, USA) and Bioanalyzer (Agilent, USA) estimated quantity and quality of each RNA sample, and the RIN (RNA Integrity Number) index ranged from 9.4 to 9.8 for all samples.

For each microarray, total RNA samples from five animals were pooled and quantity and quality of the pooled samples estimated once again by the Nanodrop and Bioanalyzer. Biotinylated cRNA was prepared using the Illumina RNA Amplification Kit (Ambion Inc., USA) according to the manufacturer's protocol and starting with 100 ng total RNA. Samples were purified with the RNeasy kit (Qiagen, Germany) according to the manufacturer's protocol.

### Microarray, Hybridization, and Fluorescent Detection 

Hybridization to the Sentrix MouseRef-8 Expression BeadChip (Illumina, USA), washing, and scanning were performed according to the Illumina BeadStation 500× manual. Sentrix MouseRef-8 Expression BeadChip contains approximately 24,000 well-annotated RefSeq 50-mer oligonucleotide probes per array. Data were extracted using software provided by the manufacturer. Illumina Beadstudio v2 software with the default settings for gene expression analysis was used.

### Data Normalization and Selection of Differentially Expressed Genes

Raw microarray data were processed with BeadArray and LIMMA package of the Bioconductor project (Bioconductor project; www.bioconductor.org). Data preprocessing step involved normalization of expression levels with quantile method was preceded by log2 transformation. Linear model fitting was performed for the pre-processed dataset. The empirical Bayes analysis was performed in order to identify differentially expressed genes by testing whether the contrast coefficients from the linear models can be assumed equal zero. Genes considered to be significantly differentially expressed with logged fold-change are greater than 0.5 and adjusted *p* value is less than 0.05. Benjamini and Hochberg method (1995), for controlling false discovery rate, was used to correct *p* values.

### Bioinformatics, Database Search, and Gene Enrichment Analysis

#### Gene Ontology Analysis

Gene lists (GenBank accession numbers) from microarray results were submitted to the Expression Analysis Systemic Explorer (EASE; http://david.abcc.ncifcrf.gov). EASE takes into account the frequencies of genes belonging to particular Gene Ontology terms (GO; http://www.geneontology.org/index.shtml) among the genes found to be regulated and among all genes studied in the experiment. EASE performs statistical analysis to detect overrepresented functional gene categories in the data set compared with all genes on the arrays. GO terms are reported with corresponding EASE scores—it is a conservative statistical test that gives the upper bound of the distribution of the Jackknife Fisher exact probabilities and favors robust categories. Functional gene categories were considered significantly overrepresented at *p* < 0.05. Genes to functional categories were classified with biological process, molecular function, and cellular component ontologies.

#### KEGG Biochemical Pathways Analysis

Analysis of the genes' association with physiological pathways was performed using the Kyoto Encyclopedia of Genes and Genomes database (KEGG; http://www.genome.jp/kegg/pathway.html). In KEGG database, distributions of the differentially expressed genes were classified among biochemical pathways. Microarray data from a single gene in a pathway do not suffice to describe a regulatory mechanism of the pathway. Therefore, only pathways with the microarray information for at least two genes were considered. To identify significantly overrepresented biological categories and KEGG pathways within the lists of differentially expressed genes, the threshold of EASE Score for the enrichment analysis was set at *p* ≤ 0.05.

#### Functional Clustering

For a more insightful view of the relationships between annotation categories compared with chart, genes were clustered in the DAVID 6.7 Functional Annotation Clustering module (http://david.abcc.ncifcrf.gov/). Biologically meaningful clusters consist of genes that are annotated into specific functional annotation groups. Grouping genes based on functional similarity can systematically enhance biological interpretation of large lists of genes derived from high throughput studies. The Functional Classification Tool generates a gene-to-gene similarity matrix based on shared functional annotation using over 75,000 terms from 14 functional annotation sources. DAVID clustering algorithms classifies highly related genes into functionally related groups. The classification stringency of the cluster analysis was set at “high” level. To avoid over-counting duplicated genes in the chart report view, the Fisher exact statistics was calculated based on corresponding DAVID gene IDs by which all redundancies in original IDs are removed.

#### Quantitative Real-Time Reverse Transcription PCR 

To validate the results of microarrays, quantitative real-time RT-PCR (qPCR) with SYBR Green technique was performed as described previously (Lisowski et al. 2011). Seven genes belonging to different functional groups and significantly differing in expression between the control and CMS mice of each strain were selected. qPCR assays were carried out in triplicates on the same but non-pooled individual RNA samples (*n* = 15) per experimental group RNA samples, which were used in the microarray experiment. For reference, two housekeeping genes, selected from ten commonly used reference genes using previous methodology (Lisowski et al. 2008a, b) stably expressed in mouse hippocampus in applied experimental assay and belonging to different functional classes, were used: glyceraldehyde-3-phosphate dehydrogenase (*Gapdh*) and hypoxanthine guanine phosphoribosyl transferase 1 (*Hprt1*). Primers were designed using ExonPrimer software (http://ihg2.helmholtz-muenchen.de/ihg/ExonPrimer.html) (Institute of Human Genetics, TUM/Helmholtz Center Munich, Germany) using *Mus musculus* GenBank sequences. All primers produced amplicons which spanned two exons each in highly conserved coding regions and included all known alternatively spliced mRNA variants

Data from three runs were calibrated by calculating the average cycle threshold value over samples in each run and the results were calculated using the mathematical model for relative quantification in qPCR described by Pfaffl (2001). To test for the effects of the observational groups on target genes mRNA level, t-test was performed (SAS version 8.02; SAS Institute, NC). Results are reported as the mean ± standard error of the mean (SEM). Differences between the groups were considered significant at *p* < 0.05. The degree of significance and the correlation between fold changes, as determined in the microarray analysis and those determined by qPCR, was evaluated with the Pearson moment correlation.

## Results

Expression profiling was designed to determine the impact of genetic background (two selectively bred strains) on the transcriptional effects of CMS in prefrontal cortex. Gene analysis was performed by one color hybridization of the 24 K microarray in HA and LA, naïve and CMS mice. Three independent biological replicates of microarray were prepared for each group of mice. To minimize the influence of individual differences between the animals and variation introduced by dissection and tissue preparation, total RNA separately isolated from several PFCs was pooled. Each pool, containing total RNA from five individuals, was separately converted to cRNA and hybridized to a single microarray.

The identified genes are considered to be expressed in the prefrontal cortex according to Novartis Gene Expression Atlas (http://www.biogps.gnf.org/). Cell-type classification analysis of differentially expressed genes, according to Cahoy (2008), GeneCards Database (http://www.genecards.org/) screening and Ingenuity Pathway Analysis (IPA) (http://www.ingenuity.com/) of top canonical pathways, revealed that most of them are characteristic for neurons and oligodendrocytes.

### Differences in Basal Gene Expression of HA Mice Compared to LA

Comparison of basal gene expression profiles between the HA and LA strains identified 193 transcripts with different levels of mRNA in the PFC that met the criteria of logged fold change greater than 0.5 and *p* < 0.05. One hundred thirty-three of differentially expressed genes between the strains were upregulated and 60 genes were downregulated in HA as compared to LA. Expression of these genes differs at baseline in a genotype-dependent manner. The whole sets of probes that differed in naive HA vs. LA mice are presented in “Electronic Supplementary Material” (ESM) Table S1 **.** Genes annotated to these probe sets were considered to be expressed in the examined brain tissue according to Novartis Gene Expression Atlas (www.biogps.gnf.org).

**Fig. 1:**
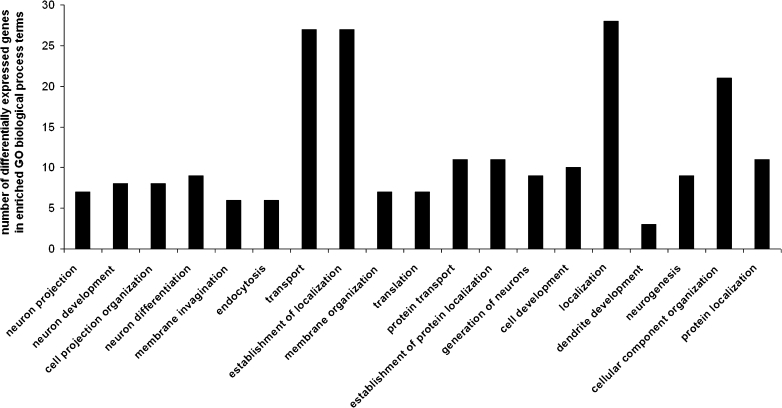
Significantly enriched (*p* < 0.05) gene ontology (GO) biological process categories of differentially expressed genes in the prefrontal cortex of naïve high analgesia (HA) vs. naïve low analgesia (LA) mice

**Fig. 2:**
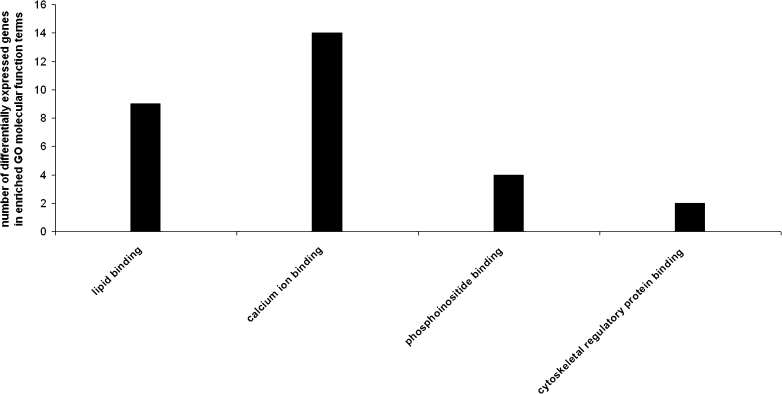
Significantly enriched (*p* < 0.05) gene ontology (GO) molecular function categories of differentially expressed genes in the prefrontal cortex of naïve high analgesia (HA) vs. naïve low analgesia (LA) mice

**Fig. 3:**
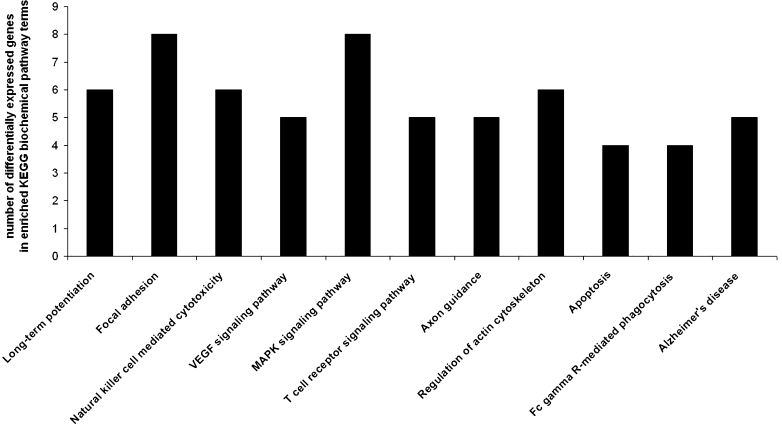
Significantly enriched (*p* < 0.05) Kyoto Encyclopedia of Genes and Genomes (KEGG) biochemical pathways of differentially expressed genes in the prefrontal cortex of naïve high analgesia (HA) vs. naïve low analgesia (LA) mice

Statistically significant (*p* < 0.05) over-expressed biological processes terms associated with the up- and downregulated genes between strains were found with the DAVID Functional Annotation Tool. We identified functional categories of biological process (Fig. 1), molecular function (Fig. 2), and KEGG biochemical pathways terms (Fig. 3), including elements of neuron development and differentiation, dendrite development, protein transport and localization, lipid binding, calcium ion binding, phosphoinositide binding, cytoskeletal regulatory protein binding, long-term potentiation, VEGF, MAPK, and/or T cell signaling pathways.

GO and KEGG classifications on a list of significant upregulated transcripts was done in each strain. In general, genes upregulated in HA encode elements involved in processes such as dendrite development (*Mtap2*, *Mtap1b*, and *Pak1*), neuron projection (*Mtap2, Mtap1b*, *Stxbp1*, *Etv1*, and *Pak1*), cell communication (*Kif1b*, *Snx14*, *Snx17*, *Clstn1*, *Stxbp1*, *Snx2*, and *Ppp3ca*), synaptic transmission (*Kif1b*, *Clstn1*, *Stxbp1*, and *Ppp3ca*), lipid binding (*Snap91*, *Dgke*, *Snx14*, *Rasgrp1*, *Snx17*, *Snx2*, *Prkcc*, and *Sh3gl2*), and calcium ion binding (*Atp2c1*, *Rasgrp1*, *Itgav*, *Clstn1*, *Cacng3*, *Kcnip2*, *Capn2*, *Prkcc*, *1500003003rik*, and *Dtna)* (Tables 1 and 2).

**Table 1:** Upregulated genes associated with the gene ontology (GO) biological process terms (*p* < 0.05) in the prefrontal cortex of high analgesia (HA) mice

GO biological process	Number of genes	EASE score	Genes
Endocytosis	6	0.002	CAV2, ITGAV, SNX17, STXBP1, SH3GL2, ELMO1
Membrane organization	7	0.002	CAV2, SNAP91, ITGAV, SNX17, STXBP1, SH3GL2, ELMO1
Organelle organization	12	0.014	TSPYL1, EPB4,1L3, CAV2, SNAP91, ATP2C1, MTAP2, MTAP1B, STXBP1, CBX3, ACIN1, RBBP7, ELMO1
Transport	20	0.016	GDI1, CAV2, SNAP91, SNX14, KCNAB1, SNX17, STXBP1, SNX2, ATP6V1G2, CACNG3, KCNIP2, ELMO1, RAB7, SLCO1A4, KIF1B, ATP2C1, ITGAV, PPP3CA, SH3GL2, SEC61A2
Dendrite development	3	0.016	MTAP2, MTAP1B, PAK1
Establishment of localization	20	0.017	GDI1, CAV2, SNAP91, SNX14, KCNAB1, SNX17, STXBP1, SNX2, ATP6V1G2, CACNG3, KCNIP2, ELMO1, RAB7, SLCO1A4, KIF1B, ATP2C1, ITGAV, PPP3CA, SH3GL2, SEC61A2
Cellular component organization	17	0.018	CAV2, SNAP91, MTAP2, SNX17, STXBP1, CBX3, RBBP7, ELMO1, TSPYL1, EPB4,1L3, ATP2C1, ITGAV, MTAP1B, ETV1, PAK1, ACIN1, SH3GL2
Neuron projection	5	0.023	MTAP2, MTAP1B, STXBP1, ETV1, PAK1
Vesicle-mediated transport	7	0.027	CAV2, SNAP91, ITGAV, SNX17, STXBP1, SH3GL2, ELMO1
Cell communication	7	0.028	KIF1B, SNX14, SNX17, CLSTN1, STXBP1, SNX2, PPP3CA
Protein transport	8	0.040	GDI1, SNX14, SNX17, STXBP1, SNX2, PPP3CA, SEC61A2, RAB7
Synaptic transmission	4	0.040	KIF1B, CLSTN1, STXBP1, PPP3CA

**Table 2:** Upregulated genes associated with the gene ontology (GO) molecular function terms (*p* < 0.05) in the prefrontal cortex of high analgesia (HA) mice

GO molecular function	Number of genes	EASE score	Genes
Lipid binding	8	0.002	SNAP91, DGKE, SNX14, RASGRP1, SNX17, SNX2, PRKCC, SH3GL2
Phosphoinositide binding	4	0.006	SNAP91, SNX14, SNX17, SNX2
Cytoskeletal regulatory protein binding	2	0.017	MTAP2, MTAP1B
Phospholipid binding	4	0.024	SNAP91, SNX14, SNX17, SNX2
Calcium ion binding	10	0.025	ATP2C1, RASGRP1, ITGAV, CLSTN1, CACNG3, KCNIP2, CAPN2, PRKCC, 1500003O03RIK, DTNA
Protein binding	39	0.026	CAV2, SNAP91, SNX14, SNX17, CLSTN1, SNX2, CBX3, ATP6V1G2, COPS8, ARPC5, FBXW7, SFRS5, CASP9, ITGAV, INSIG1, BTBD3, ZFP238, PPP3CA, PAK1, RNF14, SEC61A2, DTNA, MTAP2, EEF1A2, STXBP1, CAPN2, RBBP7, PPP1CB, ELMO1, EPB4,1L3, MAPK1, ATF4, KIF1B, BTG1, MTAP1B, LASS1, HSPD1, CD200, SH3GL2
Diacylglycerol binding	3	0.035	DGKE, RASGRP1, PRKCC

To find significantly over-represented pathways in the list of differentially expressed genes, we searched the KEGG database. In the HA mice prefrontal cortex, significantly overrepresented pathways were the long-term potentiation, focal adhesion, NK mediated cytotoxicity, VEGF, MAPK, T cell receptor, axon guidance, regulation of actin cytoskeleton, apoptosis, Fc gamma R-mediated phagocytosis and Alzheimer's disease pathways (Table 3).

**Table 3:** Upregulated genes associated with the Kyoto Encyclopedia of Genes and Genomes (KEGG) biochemical pathways (*p* < 0.05) in the prefrontal cortex of high analgesia (HA) mice

KEGG biochemical pathway	Number of genes	EASE score	Genes
Long-term potentiation	6	0.000	MAPK1, ATF4, PPP3CA, PPP1CB, PRKCC, 1500003O03RIK
Natural killer cell-mediated cytotoxicity	6	0.001	MAPK1, H2-T23, PPP3CA, PAK1, PRKCC, 1500003O03RIK
MAPK signaling pathway	8	0.001	MAPK1, ATF4, RASGRP1, CACNG3, PPP3CA, PAK1, PRKCC, 1500003O03RIK
Focal adhesion	7	0.001	MAPK1, CAV2, ITGAV, PAK1, CAPN2, PPP1CB, PRKCC
VEGF signaling pathway	5	0.001	MAPK1, CASP9, PPP3CA, PRKCC, 1500003O03RIK
T cell receptor signaling pathway	5	0.004	MAPK1, RASGRP1, PPP3CA, PAK1, 1500003O03RIK
Apoptosis	4	0.014	CASP9, PPP3CA, CAPN2, 1500003O03RIK
Alzheimer's disease	5	0.015	MAPK1, CASP9, PPP3CA, CAPN2, 1500003O03RIK
Fc gamma R-mediated phagocytosis	4	0.017	MAPK1, PAK1, ARPC5, PRKCC
Regulation of actin cytoskeleton	5	0.036	MAPK1, ITGAV, PAK1, ARPC5, PPP1CB
Axon guidance	4	0.038	MAPK1, PPP3CA, PAK1, 1500003O03RIK
Amyotrophic lateral sclerosis (ALS)	3	0.042	CASP9, PPP3CA, 1500003O03RIK

Genes upregulated in LA are involved in neuron differentiation (*Sema5a*, *Exoc7*, *Pcsk9*, and *Rpgrip1*), response to stimulus (*Crhr1*, *Bat5*, *Ercc5*, *S100a8*, *Camp*, *S100a9*, *Pcsk9*, *Mpo*, *Rpgrip1*, and *Psmb9)*, structural constituent of ribosome (*Mrps18c*, *Rpl6*, and *Rps15a)*, and coding cellular components such as cytosol and cytoplasmic parts (*Actb*, *Bat5*, *Exoc7*, *Camp*, *Rps15a*, *Psmb9*, *Mrps18c*, *Rpl6*, *Tor1b*, *Pcsk9*, *Ltf*, *Mpo*, *Neu1*, *Pdrg1*, *Tomm22*, *Slc4a1*, *Hbb-b2*, and *Srp9)*, cell cortex parts (*Actb*, *Exoc7*, and *Slc4a1*), macromolecular complex (*Bat5*, *Mrps18c*, *Kif3a*, *Exoc7*, *Rpl6*, *Rps15a*, *Pdrg1*, *Hbb-b2*, *Srp9*, *Itgbl1*, and *Psmb9)*, secretory granule and cytoplasmic membrane-bounded vesicle (*Camp*, *Mpo*, *Ltf*, and *Neu1*), and ribosome (*Mrps18c*, *Rpl6*, and *Rps15a*) (Tables 4 and 5). Among the genes upregulated in LA mice, no statistically significant KEGG biochemical pathways were found.

**Table 4:** Upregulated genes associated with the gene ontology (GO) biological process terms (*p* < 0.05) in the prefrontal cortex of low analgesia (LA) mice

GO biological process	Number of genes	EASE score	Genes
Neuron differentiation	4	0.046	SEMA5A, EXOC7, PCSK9, RPGRIP1
Response to stimulus	10	0.048	CRHR1, BAT5, ERCC5, S100A8, CAMP, S100A9, PCSK9, MPO, RPGRIP1, PSMB9
Generation of neurons	4	0.049	SEMA5A, EXOC7, PCSK9, RPGRIP1

**Table 5:** Upregulated genes associated with the gene ontology (GO) molecular function terms (*p* < 0.05) in the prefrontal cortex of low analgesia (LA) mice

GO molecular function	Number of genes	EASE score	Genes
Structural constituent of ribosome	3	0.033	MRPS18C, RPL6, RPS15A

### Effects of Chronic Mild Stress on Gene Expression in HA and LA Mice

The influence of genetic background on gene expression level was estimated. Using the same statistical criteria, the comparisons of PFC transcriptomic profiles of naive vs. CMS animals revealed 96 in HA and 92 in LA differentially expressed transcripts. In HA strain, 59 of differentially expressed were upregulated and 37 genes were downregulated after CMS (ESM Table S2), while in LA strain, 60 genes were upregulated and 32 were downregulated as a result of CMS (ESM Table S3). There was also some overlap in the expression profiles between the strains: 23 common transcripts were changed in both strains (Table 6). Within the list of the overlapping genes, according to DAVID Functional Clustering Tool, eight genes coded cell membrane parts (*Rab5b*, *Cntnap4*, *Ai593442*, *Pigt*, *Tomm22*, *Rgs9*, *Rasd2*, and *Calb2*), six genes were classified into signal transduction and intracellular signaling cascade cluster (*Rab5b*, *Cntnap4*, *Dgkg*, *Rgs9*, *Mtss1l*, and *Rasd2)*, three genes into ion binding cluster (*Dgkg*, *Nell2*, and *Calb2*), and three genes into transport and establishment and localization cluster (*Ttr*, *Rab5b*, and *Tomm22*) (Table 7).

**Table 6:** Common transcripts with overlapping changes in the prefrontal cortex of high (HA) and low (LA) analgesia mice followed chronic mild stress (CMS)

Gene symbol	Definition	Expression	HA fold change	LA fold change
Ttr	Transthyretin (Ttr)	↑	6.09	2.54
Tomm22	Translocase of outer mitochondrial membrane 22	↑↓	2.75	−2.18
C1ql2	Complement component 1, q subcomponent-like 2	↑	1.32	1.48
1300006M19Rik	RIKEN cDNA 1300006 M19 gene	↑	2.26	2.38
0610009K11Rik	Mitochondrial ubiquitin ligase activator of NFKB 1	↑↓	2.12	−1.58
Wdr6	WD repeat domain 6 (Wdr6)	↑	1.97	1.56
Mtss1l	Metastasis suppressor 1-like	↑	1.45	1.55
BC040774		↑	2.46	2.20
BC060632		↑	1.86	1.65
Nell2	Nel-like 2 homolog (chicken) (Nell2)	↑	1.54	2.29
Calb2	calbindin 2 (Calb2)	↑	1.69	1.29
Rab5b	RAB5B, member RAS oncogene family (Rab5b)	↑	1.58	2.36
Krt1-12	Keratin complex 1, acidic, gene 12 (Krt1-12)	↑↓	1.61	−1.37
Nnat	Neuronatin (Nnat), transcript variant 2	↑	1.50	1.83
Dgkg	Diacylglycerol kinase, gamma (Dgkg)	↑	1.30	1.25
C630041L24Rik	RIKEN cDNA C630041L24 gene	↑	1.28	1.35
Cntnap4	Contactin-associated protein 4 (Cntnap4)	↑	0.52	0.75
Ddx6	DEAD (Asp-Glu-Ala-Asp) box polypeptide 6	↓	−1.38	−1.46
AI593442	*Mus musculus* expressed sequence AI593442	↓	−1.51	−1.68
Rasd2	RASD family, member 2	↓	−1.73	−1.64
Indo	Indoleamine-pyrrole 2,3 dioxygenase	↓	−2.19	−1.89
Rgs9	Regulator of G-protein signaling 9 (Rgs9)	↓	−2.35	−2.31
Pigt	Phosphatidylinositol glycan, class T (Pigt)	↓	−3.22	−1.25

“↑” upregulation, “↓” downregulation

**Table 7:** Clusters of overlapping transcripts determined by the DAVID v6.7 software (*p* < 0.05)

Gene symbol	Definition
Signal transduction/intracellular signaling cascade	
Rab5b	RAB5B, member RAS oncogene family
Rasd2	RASD family, member 2
Cntnap4	Contactin-associated protein-like 4
Dgkg	Diacylglycerol kinase, gamma
Mtss1l	Metastasis suppressor 1-like
Rgs9	Regulator of G-protein signaling 9
Calcium ion binding/metal ion binding/cation binding	
Nell2	NEL-like 2 (chicken)
Calb2	Calbindin 2
Dgkg	Diacylglycerol kinase, gamma
Transport/establishment of localization	
Rab5b	RAB5B, member RAS oncogene family
Tomm22	Translocase of outer mitochondrial membrane 22 homolog
Ttr	Transthyretin
Membrane part	
Rab5b	RAB5B, member RAS oncogene family
Rasd2	RASD family, member 2
Calb2	Calbindin 2
Cntnap4	Contactin-associated protein-like 4
AI593442	Expressed sequence AI593442
Pigt	Phosphatidylinositol glycan anchor biosynthesis
Tomm22	Translocase of outer mitochondrial membrane 22 homolog
Rgs9	Regulator of G-protein signaling 9

**Fig. 4:**
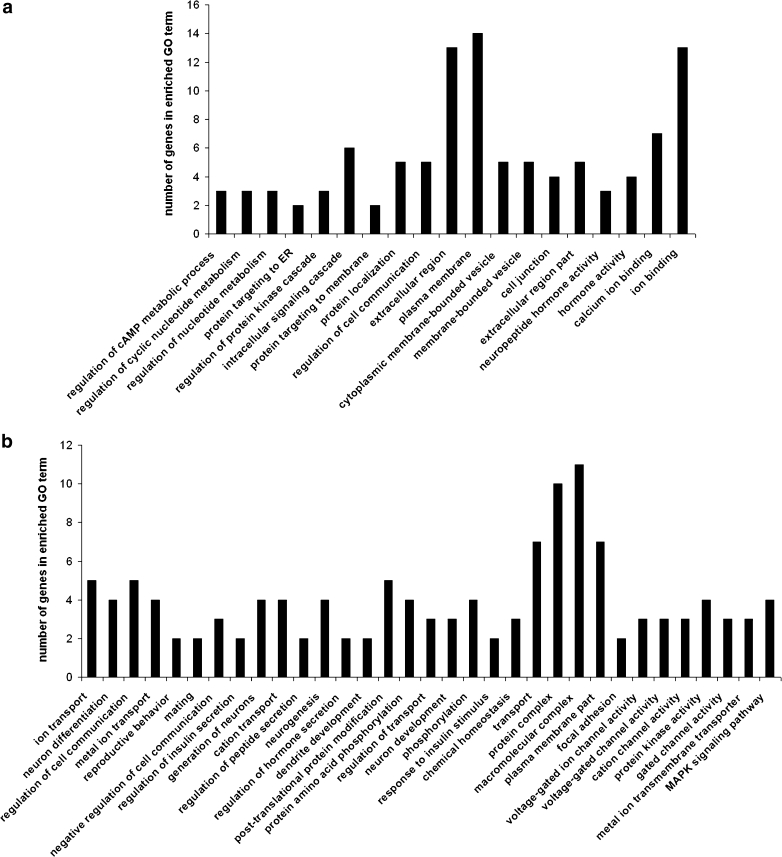
Significantly enriched (*p* < 0.05) gene ontology (GO) categories of genes affected by chronic mild stress (CMS) in the prefrontal cortex of high analgesia (HA) mice: **a** upregulated transcripts, and **b** downregulated transcripts. GO categories shown in the figure consists of biological processes, molecular functions, cellular components, and biochemical pathways

Statistically significant (*p* < 0.05) over-expressed terms associated with the up- and downregulated genes were found in both strains subjected to CMS. Functional annotation was done in each strain on a list of up- and downregulated transcripts separately. In HA mice, CMS affected the upregulation of genes involved in, e.g., intracellular signaling, ion binding, neuropeptide hormone activity, and metabolism of cAMP and nucleotides (Fig. 4a). Downregulated genes were those involved in ion transport, reproductive and mating behavior, neuron differentiation and dendrite development, cell communication, regulation of insulin secretion and response to insulin stimulus, regulation of transport, homeostasis, focal adhesion, ion channel activity, or MAPK signaling pathway (Fig. 4b).

**Fig. 5:**
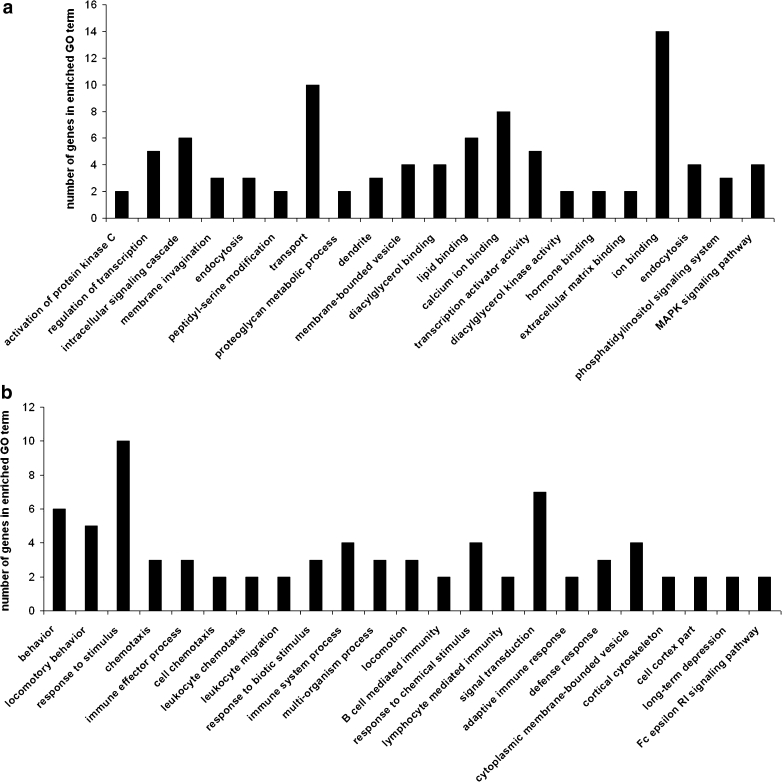
Significantly enriched (*p* < 0.05) gene ontology (GO) categories of genes affected by chronic mild stress (CMS) in the prefrontal cortex of low analgesia (LA) mice: **a** upregulated transcripts, and **b** downregulated transcripts. GO categories shown in the figure consists of biological processes, molecular functions, cellular components and biochemical pathways

In LA mice, CMS affected the upregulation of genes involved in the activation of protein kinase C, regulation of transcription, calcium ion binding, hormone binding, or coding elements of dendrites (Fig. 5a). Downregulated genes were involved in locomotory behavior, signal transduction and immunity processes, or long-term depression (Fig. 5b).

For more detailed data interpretation, we performed the functional clustering of DEGs. Functional clustering of DEGs between stressed vs. control animals revealed several functional groups of genes in each strain. Altogether, 11 main clusters were found for HA (Table 8) and 12 for LA (Table 9). Specific clusters for HA strain contain genes involved in regulation of hormone secretion/regulation of insulin secretion, ion binding, regulation of primary metabolic process/regulation of cAMP biosynthetic process, and cell junction/adherens junction. Specific clusters for LA strain contain genes involved in response to stress, dendrite/neuron projection, immune effector process/immune response, intracellular signaling cascade/GTPase regulator activity and cognition. Functional clustering revealed similar clusters in both lines despite containing different genes. Overlapping clusters in both strains were clusters such as signal transduction, response to stimulus, regulation of biological quality and homeostasis, cell differentiation, apoptosis and cell death, regulation of transcription and gene expression, and signal transducer/receptor activity. Clusters were identified in the list of the significantly upregulated and downregulated genes in non stressed vs*.* stressed animals. Tables 8 and 9 present significant functional clusters with differentially expressed genes and their expression values.

**Table 8:** Clusters of genes determined by the DAVID v6.7 software (*p* < 0.05) affected by chronic mild stress (CMS) in the prefrontal cortex of high analgesia (HA) mice

Gene symboal	Gene name	Expression	Fold Change
Regulation of cell communication/regulation of signal transduction			
Bat2	HLA-B-associated transcript 2	↑	1.48
Acvr1c	Activin A receptor, type IC	↓	−2.10
Bai1	Brain-specific angiogenesis inhibitor 1	↑	1.61
Dkk3	Dickkopf homolog 3 (Xenopus laevis)	↓	−1.48
Drd1a	Dopamine receptor D1A	↓	−1.28
Mtap1b	Microtubule-associated protein 1B	↓	−1.45
Palm	Paralemmin	↑	1.34
Cbx3	Predicted gene 6917; similar to chromobox homolog 3	↓	−2.17
Rgs9	Regulator of G-protein signaling 9	↓	−2.35
Sostdc1	Sclerostin domain containing 1	↑	1.50
Srp9	Signal recognition particle 9	↑	2.49
Timp2	Tissue inhibitor of metalloproteinase 2	↑	1.55
Regulation of hormone secretion/regulation of insulin secretion			
Bat2	HLA-B-associated transcript 2	↑	1.48
Acvr1c	Activin A receptor, type IC	↓	−2.10
Avp	Arginine vasopressin	↑	3.67
Drd1a	Dopamine receptor D1A	↓	−1.28
Il12a	Interleukin 12a	↓	−1.32
Kl	Klotho	↓	−2.20
Nnat	Neuronatin	↑	1.50
Pfkm	Phosphofructokinase, muscle	↓	−1.83
Ion binding			
Arl3	ADP-ribosylation factor-like 3	↑	3.10
Lasp1	LIM and SH3 protein 1	↓	−1.85
Nell2	NEL-like 2 (chicken)	↑	1.70
Acvr1c	Activin A receptor, type IC	↓	−2.10
Calb2	Calbindin 2	↑	1.69
Cacna2d1	Calcium channel, voltage-dependent, alpha2/delta subunit 1	↓	−1.38
Calml4	Calmodulin-like 4	↑	1.52
Clic6	Chloride intracellular channel 6	↑	1.77
Dgkg	Diacylglycerol kinase, gamma	↑	1.30
Hpcal4	Hippocalcin-like 4	↑	1.22
Kl	Klotho	↑	1.92
Mmp17	Matrix metallopeptidase 17	↓	−1.26
Myl4	Myosin, light polypeptide 4	↓	−1.50
Pfkm	Phosphofructokinase, muscle	↓	−1.83
Kcnb1	Potassium voltage gated channel, Shab-related subfamily	↓	−1.98
Kcnh1	Potassium voltage-gated channel, subfamily H (eag-related)	↓	−1.85
Pnck	Pregnancy upregulated non-ubiquitously expressed CaM kinase	↑	1.40
Prkcb	Protein kinase C, beta	↓	−0.65
Sparc	Secreted acidic cysteine rich glycoprotein	↑	1.54
Slc17a7	Solute carrier family 17	↑	1.22
Zfp423	Zinc finger protein 423; similar to mKIAA0760 protein	↑	1.42
Zcchc12	Zinc finger, CCHC domain containing 12	↑	1.89
Regulation of primary metabolic process/regulation of cAMP biosynthetic process			
Avp	Arginine vasopressin	↑	3.67
Drd1a	Dopamine receptor D1A	↓	−1.28
Palm	Paralemmin	↑	1.34
Kcnh1	Potassium voltage-gated channel, subfamily H	↓	−1.85
Cbx3	Predicted gene 6917; similar to chromobox homolog 3	↓	−2.17
Rbbp4	Retinoblastoma binding protein 4	↓	−1.87
Srp9	Signal recognition particle 9	↑	2.49
Timp2	Tissue inhibitor of metalloproteinase 2	↑	1.55
Zfp423	Zinc finger protein 423; similar to mKIAA0760 protein	↑	1.42
Zcchc12	Zinc finger, CCHC domain containing 12	↑	1.89
Cell junction/adherens junction			
Lasp1	LIM and SH3 protein 1	↓	−1.85
Calb2	Calbindin 2	↑	1.69
Cbln1	Cerebellin 1 precursor protein; similar to precerebellin-1	↓	−2.06
Pak1	p21 protein (Cdc42/Rac)-activated kinase 1	↓	−2.06
Pkp2	Plakophilin 2	↑	1.26
Slc17a7	Solute carrier family 17	↑	1.22
Response to endogenous stimulus/response to hormone stimulus			
Bat2	HLA-B-associated transcript 2	↑	1.48
Bat5	HLA-B-associated transcript 5	↑	3.36
Rasd2	RASD family, member 2	↓	−1.73
Acvr1c	Activin A receptor, type IC	↓	−2.10
Avp	Arginine vasopressin	↑	3.67
Drd1a	Dopamine receptor D1A	↓	−1.28
H2-L	Histocompatibility 2, D region; histocompatibility 2	↑	2.56
Il12a	Interleukin 12a	↓	−1.32
Nnat	Neuronatin	↑	1.50
Ppp1r1b	Protein phosphatase 1, regulatory (inhibitor) subunit 1B	↓	−1.68
Ptpra	Protein tyrosine phosphatase, receptor type, A	↓	−1.78
Rgs9	Regulator of G-protein signaling 9	↓	−2.35
Slc17a7	Solute carrier family 17	↑	1.22
Trh	Thyrotropin-releasing hormone	↑	1.91
Homeostatic process/regulation of biological quality			
Avp	Arginine vasopressin	↑	3.67
Drd1a	Dopamine receptor D1A	↓	−1.28
Nts	Neurotensin	↑	1.58
Palm	Paralemmin	↑	1.34
Pfkm	Phosphofructokinase, muscle	↓	−1.83
Prkcb	Protein kinase C, beta	↓	−0.65
Ppp1r1b	Protein phosphatase 1, regulatory (inhibitor) subunit 1B	↓	−1.68
Slc17a7	Solute carrier family 17	↑	1.22
Generation of neurons/neurogenesis/neuron differentiation			
Acvr1c	Activin A receptor, type IC	↓	−2.10
Dkk3	Dickkopf homolog 3 (Xenopus laevis)	↓	−1.48
Drd1a	Dopamine receptor D1A	↓	−1.28
Kl	Klotho	↑	1.92
Mtap1b	Microtubule-associated protein 1B	↓	−1.45
Nnat	Neuronatin	↑	1.50
Pak1	p21 protein (Cdc42/Rac)-activated kinase 1	↓	−2.06
Pigt	Phosphatidylinositol glycan anchor biosynthesis, class T	↓	−3.22
Pkp2	Plakophilin 2	↑	1.26
Prlr	Prolactin receptor	↑	1.62
Sostdc1	Sclerostin domain containing 1	↑	1.50
Timp2	Tissue inhibitor of metalloproteinase 2	↑	1.55
Zfp423	Zinc finger protein 423; similar to mKIAA0760 protein	↑	1.42
Apoptosis/cell death			
Bat2	HLA-B-associated transcript 2	↑	1.48
Acvr1c	Activin A receptor, type IC	↓	−2.10
Pigt	Phosphatidylinositol glycan anchor biosynthesis, class T	↓	−3.22
Regulation of gene expression/regulation of transcription			
Kcnh1	Potassium voltage-gated channel, subfamily H	↓	−1.85
Cbx3	Predicted gene 6917; similar to chromobox homolog 3	↓	−2.17
Rbbp4	Retinoblastoma binding protein 4	↓	−1.87
Srp9	Signal recognition particle 9	↑	2.49
Zfp423	Zinc finger protein 423; similar to mKIAA0760 protein	↑	1.42
Zcchc12	Zinc finger, CCHC domain containing 12	↑	1.89
Signal transducer activity/receptor activity			
Acvr1c	Activin A receptor, type IC	↓	−2.10
Bai1	Brain-specific angiogenesis inhibitor 1	↑	1.61
Drd1a	Dopamine receptor D1A	↓	−1.28
Folr1	Folate receptor 1 (adult)	↑	1.23
Igsf1	Immunoglobulin superfamily, member 1	↑	2.09
Kcnh1	Potassium voltage-gated channel, subfamily H	↓	−1.85
Tomm22	Predicted gene 12906; predicted gene 7250	↑	2.75
Prlr	Prolactin receptor	↑	1.62
Ptpra	Protein tyrosine phosphatase, receptor type, A	↓	−1.78
Rgs9	Regulator of G-protein signaling 9	↓	−2.35

“↑” upregulation, “↓” downregulation

**Table 9:** Clusters of genes determined by the DAVID v6.7 software (*p* < 0.05) affected by chronic mild stress (CMS) in the prefrontal cortex of low analgesia (LA) mice

Gene symbol	Gene name	Expression	Fold change
Signal transduction			
Fcer1g	Fc receptor, IgE, high affinity I, gamma polypeptide	↓	−2.66
Gpr88	G-protein coupled receptor 88	↓	−1.23
Rab5b	RAB5B, member RAS oncogene family	↑	2.36
Rasgrp1	RAS guanyl-releasing protein 1	↑	1.31
Rasd2	RASD family, member 2	↓	−1.64
Cntnap4	Contactin-associated protein-like 4	↑	0.75
Crhr1	Corticotropin-releasing hormone receptor 1	↓	−1.76
Dgke	Diacylglycerol kinase, epsilon	↑	1.66
Dgkg	Diacylglycerol kinase, gamma	↑	1.25
Grm5	Glutamate receptor, metabotropic 5	↓	−0.95
Gdi1	Guanosine diphosphate (GDP) dissociation inhibitor 1	↑	1.99
Matk	Megakaryocyte-associated tyrosine kinase	↓	−1.53
Prkcc	Protein kinase C, gamma	↑	2.54
Rgs9	Regulator of G-protein signaling 9	↓	−2.31
Spock2	Sparc/osteonectin, cwcv and kazal-like domains proteoglycan 2	↑	1.44
Response to stress			
Fcer1g	Fc receptor, IgE, high affinity I, gamma polypeptide	↓	−2.66
Rasd2	RASD family, member 2	↓	−1.64
S100a8	S100 calcium binding protein A8 (calgranulin A)	↓	−3.78
S100a9	S100 calcium binding protein A9 (calgranulin B)	↓	−3.32
Capn2	Calpain 2	↑	3.76
Camp	Cathelicidin antimicrobial peptide	↓	−1.72
Crhr1	Corticotrophin-releasing hormone receptor 1	↓	−1.76
Grm5	Glutamate receptor, metabotropic 5	↓	−0.95
Mt3	Metallothionein 3	↑	1.89
Mpo	Myeloperoxidase	↓	−1.85
Nnat	Neuronatin	↑	1.83
Igh-VJ558	Predicted gene 5353; immunoglobulin heavy chain (J558 family)	↓	−1.63
Prkcc	Protein kinase C, gamma	↑	2.54
Rgs9	Regulator of G-protein signaling 9	↓	−2.31
Dendrite/neuron projection			
Crmp1	Collapsin response mediator protein 1	↑	1.36
Cntnap4	Contactin-associated protein-like 4	↑	0.75
Prkcc	Protein kinase C, gamma	↑	2.54
Immune effector process/immune response			
Atp6v0a1	ATPase, H + transporting, lysosomal V0 subunit A1	↑	1.62
Fcer1g	Fc receptor, IgE, high affinity I, gamma polypeptide	↓	−2.66
S100a9	S100 calcium binding protein A9 (calgranulin B)	↓	−3.32
Mpo	Myeloperoxidase	↓	−1.85
Igh-VJ558	Predicted gene 5353; immunoglobulin heavy chain (J558 family)	↓	−1.63
Intracellular signaling cascade/GTPase regulator activity			
Rab5b	RAB5B, member RAS oncogene family	↑	2.36
Rasgrp1	RAS guanyl-releasing protein 1	↑	1.31
Rasd2	RASD family, member 2	↓	−1.64
Dgke	Diacylglycerol kinase, epsilon	↑	1.66
Dgkg	Diacylglycerol kinase, gamma	↑	1.25
Gdi1	Guanosine diphosphate (GDP) dissociation inhibitor 1	↑	1.99
Prkcc	Protein kinase C, gamma	↑	2.54
Rgs9	Regulator of G-protein signaling 9	↓	−2.31
Behavior/response to external stimulus			
Fcer1g	Fc receptor, IgE, high affinity I, gamma polypeptide	↓	−2.66
Rasd2	RASD family, member 2	↓	−1.64
S100a8	S100 calcium binding protein A8 (calgranulin A)	↓	−3.78
S100a9	S100 calcium binding protein A9 (calgranulin B)	↓	−3.32
Crhr1	Corticotrophin-releasing hormone receptor 1	↓	−1.76
Grm5	Glutamate receptor, metabotropic 5	↓	−0.95
Prkcc	Protein kinase C, gamma	↑	2.54
Ion homeostasis/regulation of biological quality			
Crym	Crystallin, mu	↑	1.85
Grm5	Glutamate receptor, metabotropic 5	↓	−0.95
Ltf	Lactotransferrin	↓	−2.59
Mt3	Metallothionein 3	↑	1.89
Sepw1	Selenoprotein W, muscle 1	↑	1.39
Slc26a4	Solute carrier family 26, member 4	↑	1.32
Nervous system development/cell differentiation			
Atp6v0a1	ATPase, H + transporting, lysosomal V0 subunit A1	↑	1.62
Capn2	Calpain 2	↑	3.76
Grm5	Glutamate receptor, metabotropic 5	↓	−0.95
Kif3a	Kinesin family member 3A	↓	−1.97
Lbh	Limb-bud and heart	↑	1.50
Mt3	Metallothionein 3	↑	1.89
Mog	Myelin oligodendrocyte glycoprotein	↑	1.37
Nnat	Neuronatin	↑	1.83
Nfib	Nuclear factor I/B	↑	1.37
Pigt	Phosphatidylinositol glycan anchor biosynthesis, class T	↓	−1.25
Prkcc	Protein kinase C, gamma	↑	2.54
Rgs9	Regulator of G-protein signaling 9	↓	−2.31
Slc26a4	Solute carrier family 26, member 4	↑	1.32
Thra	Thyroid hormone receptor alpha; similar to thyroid hormone receptor	↑	1.31
Regulation of apoptosis/regulation of programmed cell death			
Fcer1g	Fc receptor, IgE, high affinity I, gamma polypeptide	↓	−2.66
Eef1a2	Eukaryotic translation elongation factor 1 alpha 2	↑	1.90
Mal	Myelin and lymphocyte protein, T-cell differentiation protein	↑	1.37
Regulation of transcription/regulation of gene expression			
Atp6v0a1	ATPase, H + transporting, lysosomal V0 subunit A1	↑	1.62
Atf4	Activating transcription factor 4	↑	1.45
Brms1l	Breast cancer metastasis-suppressor 1-like	↑	1.33
Eef1a2	Eukaryotic translation elongation factor 1 alpha 2	↑	1.90
Lbh	Limb-bud and heart	↑	1.50
Nfib	Nuclear factor I/B	↑	1.37
Thra	Thyroid hormone receptor alpha; similar to thyroid hormone receptor	↑	1.31
Zfp238	Zinc finger protein 238	↑	1.81
Signal transducer activity/receptor activity/G-protein coupled receptor activity			
Fcer1g	Fc receptor, IgE, high affinity I, gamma polypeptide	↓	−2.66
Gpr88	G-protein coupled receptor 88	↓	−1.23
Crhr1	Corticotrophin-releasing hormone receptor 1	↓	−1.76
Grm5	Glutamate receptor, metabotropic 5	↓	−0.95
Mog	Myelin oligodendrocyte glycoprotein	↑	1.37
Tomm22	Predicted gene 12906; predicted gene 7250	↓	−2.18
Rgs9	Regulator of G-protein signaling 9	↓	−2.31
Thra	Thyroid hormone receptor alpha; similar to thyroid hormone receptor	↑	1.31
Cognition			
Grm5	Glutamate receptor, metabotropic 5	↓	−0.95
Prkcc	Protein kinase C, gamma	↑	2.54
Rgs9	Regulator of G-protein signaling 9	↓	−2.31

“↑” upregulation, “↓” downregulation

### Validation of Microarrays

**Fig. 6:**
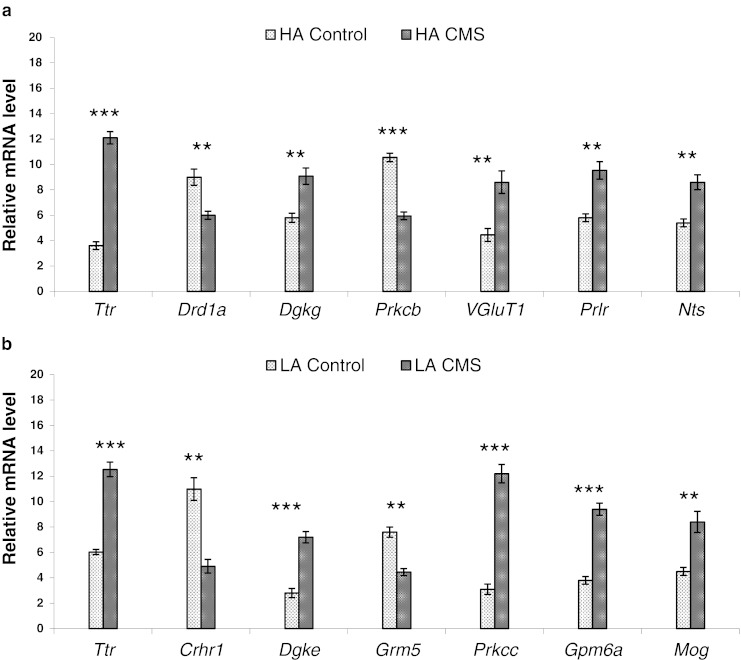
Validation of expression of the selected genes by qPCR: **a** HA control vs. HA followed by CMS; **b** LA control vs. LA followed by CMS. Results are presented as means of relative mRNA levels in 15 individuals per experimental group; error indicators show ± S.E.M. Values differ significantly at * *p* < 0.05, ** *p* < 0.01 or *** *p* < 0.001; qPCR values were normalized to geometric mean of the raw expression data of two reference genes: *Gapdh* and *Hprt1*. Abbreviations: *qPCR*, quantitative real-time RT-PCR; *HA*, high analgesia mice; *LA*, low analgesia mice; *CMS*, chronic mild stress; *S.E.M.*, standard error of measurement or mean; *Gapdh*, glyceraldehyde-3-phosphate dehydrogenase; *Hprt1*, hypoxanthine phosphoribosyltransferase 1

To confirm differential expression indicated by the microarray expression patterns, the quantitative real-time reverse transcription PCR was used (qPCR). qPCR were performed using aliquots of the non-pooled total RNA. Following genes were selected from the list of the significant functional clusters in each strain: *Ttr*, *Drd1*, *Dgkg*, *Prkcb*, *VGluT1*, *Prlr*, and *Nts* for HA strain, and *Ttr*, *Crhr1*, *Dgke*, *Grm5*, *Prkcc*, *Gpm6a*, and *Mog* for LA strain. The expression patterns of the selected genes are shown in Fig. 6a, b. Similar trends in gene expression were shown as in the microarrays. Genes selected according to microarray analyses showed corresponding values with a high correlation. Correlation for the selected data points was *R* = 0.91 (*p* < 0.0001).

## Discussion

Environmental factors and genes contribute to the manifestation of phenotype which, however, may occur or not. The proper activity of a complex organ such as the brain relies on precise temporal and spatial gene expression patterns. The phenotype is a result of a cascade of transcriptional events that finally triggers gene expression and determines the function of the particular brain structures. While a certain set of genes is constantly expressed to maintain the organ structure, a varying number of genes are regulated according to the metabolic demand of the surrounding organism. PFC is the brain region that is most sensitive to the detrimental effects of stress exposure. Abilities of PFC depend on proper neuronal network connections, which are highly sensitive to their neurochemical environment (Arnsten 2009). Since exposure to even a mild uncontrollable stress can rapidly impair PFC functions, which contribute to PFC deficits and structural changes in human and animals, a good understanding of the genetic control of chronic stress exposure will facilitate further progress in understanding the pathophysiology of stress. Gene expression level could thus be a biomarker and provide information, for example, for psychiatric diagnosis.

It is important to have animal models of stress response to understand the mechanisms that render individuals vulnerable. In this study, we have focused on the comparison of prefrontal cortex transcription profiles between naive and stressed inbred mice strains with different sensitivity to stress. Studies of differences in gene expression were carried out with 24 K oligonucleotide microarrays for genome-wide gene expression analysis and the real-time RT-PCR technique for microarray validation. To determine the physiological processes that are differentially regulated between strains, the DAVID 6.7 Functional Annotation Tool was used.

For validation of oligonucleotide chip data, we performed real-time RT-PCR with the same RNA samples that was used for the microarray experiments. *Gapdh* and *Hprt1* were used as a reference. These genes were not regulated between HA and LA during stress procedure in our study and have been used successfully as a housekeeping control in previous study as well (Lisowski et al*.* 2011). Other housekeeping genes that have been proposed such as beta actin were found to be regulated in brain between strains and/or stressed vs. control mice. In this study, the average fold change of correlation between microarray and qRT-PCR was 0.91 (*p* < 0.0001). Large-scale study of real-time RT-PCR and gene expression measurements with commercial oligonucleotide microarrays concluded that microarrays are invaluable discovery tools with acceptable reliability for genome-wide gene expression screening, though validation of putative changes in gene expression remains advisable (Wang et al*.* 2006).

### Differences in Basal Gene Expression of HA Mice Compared to LA Mice

As a way of identifying stress-responsive candidate genes, we conducted gene expression profiling studies in the PFC of high (HA) and low (LA) swim stress-induced analgesia inbred mouse strains. The strains display robust differences in behavioral tests reflecting depression and in responses to different classes of antidepressants (desipramine, venlafaxine, and aminosenktide) (Błaszczyk et al*.* 2000; Juszczak et al*.* 2008a, b, 2006; Panocka et al*.* 1986a). Experimental models of SIA help in identifying the fundamental mechanisms of stress response. The phenomenon that pain can be naturally suppressed leads to speculation that manipulation of the mechanisms of SIA could be a potential therapeutic target for stress- and anxiety-related disorders (Butler and Finn 2009). It may also be possible to utilize the expression of SIA as a predictor for development of psychological disorders (Meeus et al*.* 2008; Nishith et al*.* 2002; Pielsticker et al*.* 2005; Staud et al*.* 2003).

Identification of 193 differentially expressed genes between the strains supported by functional classification showed that selective breeding seriously affected frontal cortex development in aspect of structural changes such as neuron projection, neuron development, neuron differentiation, membrane organization, or dendrite development. SIA is mediated by activation of the descending inhibitory pain pathway that originates in neurons in higher brain regions such as the cortex. Neurons in the cortex also relay nociceptive information to the amygdala, hypothalamus, or directly to the periaqueductal grey. The PFC has also been implicated in the recall and extinction of fear-related memory of noxious stimuli (Hugues et al*.* 2004). Upregulation in the PFC of HA mice expression pattern of genes involved in calcium ion binding, lipid binding, diacyroglicerol binding, VEGF signaling, MAPK signaling, T cell receptor signaling, focal adhesion, or long-term potentiation suggests increased activation of neurons, leading to high analgesic phenotype and complex prefrontal cortex mechanisms of pain, stress, or fear.

### Effects of Chronic Mild Stress on Gene Expression in HA and LA Mice

The chronic mild stress has been studied at the cellular level (Airan et al*.* 2007; Banasr and Duman 2007; Garcia-Garcia et al. 2009; Gronli et al*.* 2007; Jayatissa et al*.* 2008; Warner-Schmidt and Duman 2006). In addition to core symptoms of depression, such as long-lasting anhedonia (Elizalde et al*.* 2008), CMS induces neuroadaptive changes that could be addressing clinical findings with depressed patients (Frodl et al. 2008; Gould et al*.* 2007; Lucassen et al*.* 2006; Rajkowska 2000; Sanacora et al*.* 2004). Very little is known about the effects of chronic stress on transcriptome in the prefrontal cortex. Recent studies on primates demonstrate that social stress reduces the expression of many genes in the PFC that are involved in synaptic plasticity, cell cycle progression, and nuclear receptor signaling (Karssen et al*.* 2007). According to Tordera (2011), CMS affected the regulation of 147 transcripts in a mouse CMS model, some of them involved in response to stress and oxidoreductase activity. Here, we aimed to identify in animals with different genetic background (HA CMS and LA CMS mice) specific gene expression profiles and common gene expression changes. We have identified genes similarly regulated by CMS in both strains. Overlapping genes with the same expression pattern in both mouse models are involved in coding elements of cell membrane part and are involved in signal transduction, calcium ion binding, and transport. We observed robust upregulation of *Ttr* gene coding transthyretin (TTR). Transthyretin is one of the three prealbumins: alpha-1-antitrypsin, transthyretin, and orosomucoid. Transthyretin is a carrier protein and a major transporter of thyroid hormones and retinol in the plasma and cerebrospinal fluid (Landers et al. 2009). The diseases caused by mutations in *TTR* include amyloidotic polyneuropathy, euthyroid hyperthyroxinaemia, amyloidotic vitreous opacities, cardiomyopathy, oculoleptomeningeal amyloidosis, meningocerebrovascular amyloidosis, or carpal tunnel syndrome (Blevins et al*.* 2003; Garzuly et al*.* 1996; Jones et al*.* 1992; Murakami et al*.* 1994, 1992; Saraiva et al. 1992). Clinical features of defects in *TTR* include seizures, stroke-like episodes, dementia, and psychomotor deterioration. The absence of TTR protein in TTR-null mice is associated with increased exploratory activity and reduced depression-like behavior (Sousa et al*.* 2004). Cell cultures incubated with TTR oligomers were proven to induce cytotoxicity by Ca^2+^ efflux from the endoplasmic reticulum (Teixeira et al. 2006). Moreover, recent observation conducted by Andrus (2012) classified *Ttr* to the set of genes whose hippocampal or amygdalar expression patterns were altered by chronic stress in four rat strains represent a generalizable molecular response to chronic stress. Taken together, we observed stress-associated significant cortical upregulation of *Ttr* and calcium ion binding genes (*Nell2*, *Calb2*, *Dgkg*) in both high and low analgesia mouse strains.

Given the genetic heterogeneity, it is likely that not all the subjects will show identical changes in all genes. We found that an individual's genetic make-up does make a serious difference in how the individual, at the gene expression level, responds to stress. Functional clustering revealed seven statistically significant overlapping groups of differentially expressed genes between control and stressed animals. Common clusters, in both strains, included genes involved in behavior, signal transduction, response to hormone stimulus, homeostasis, neuron differentiation, neurogenesis, and apoptosis. The HA strain-specific groups of genes were involved in regulation of insulin secretion, cAMP biosynthetic process and cell junction. The LA strain groups of genes were connected with response to stress, dendrite and neuron projection, immune response, GTP-ase activity, and cognition.

Exposure to chronic stress leads to extensive alterations in the architecture of PFC including loss of dendritic material (Brown et al*.* 2005). Whereas structural changes in the hippocampus require several weeks of stress exposure, dendrites in the PFC begin to change after 1 week of stress or possibly even a single exposure (Brown et al*.* 2005; Izquierdo et al. 2006; McEwen 2004). The signaling mechanisms that underlie these changes in the PFC are just beginning to be studied. Each member of the PKC family has a specific expression profile and is believed to play distinct roles in cells. Protein kinase C gamma type is expressed solely in the brain and spinal cord and its localization is restricted to neurons. It has been demonstrated that several neuronal functions, including long-term potentiation (LTP) and long-term depression, specifically require this kinase. Knockout studies in mice also suggest that this kinase may be involved in neuropathic pain development (Malmberg et al*.* 1997). In our study, we have found that CMS decreased transcription of protein kinase C, beta (*Prkcc*) in HA mice and increased protein kinase C, gamma (*Prkcg*) in LA. Glucocorticoid release during stress could potentiate stress-signaling pathways through activation of PKC signaling (Han et al*.* 2002).

Chronic stress alters catecholamine pathways increasing noradrenergic innervation of the PFC although dopamine becomes depleted with severe chronic stress (Miner et al. 2006; Mizoguchi et al. 2000). Increased noradrenaline might lead to higher levels of PKC and cAMP signaling (Arnsten 2009). In LA mice, we have identified a cluster of genes downregulated by CMS which are involved in the cAMP biosynthetic process including the dopamine receptor D1A (*Drd1a*). Moreover, PKCs require Ca^2+^, diacylglycerol (DAG), and a phospholipid such as phosphatidylserine for activation. According to this, another interesting group of genes identified in LA mice was intracellular signaling cascade/GTP-ase regulator activity cluster. This cluster, besides *Prkcg* consist of several upregulated genes in LA CMS animals including diacylglycerol kinases epsilon (*Dgke*) and gamma (*Dgkg*) involved in PKC signaling.

Stress-induced changes in dendritic morphology may be associated with upregulation of another two genes constituting dendrite/neurite projection gene cluster such as *Crmp1* (collapsin response mediator protein 1) and *Cntnap4* (contactin associated protein-like 4). *Crmp1* encodes a member of a family of cytosolic phosphoproteins expressed exclusively in the nervous system. Encoded protein is thought to be a part of the semaphorin signal transduction pathway implicated in semaphorin-induced growth cone collapse. CRMP1 is necessary for signaling by class 3 semaphorins and subsequent remodeling of the cytoskeleton. Furthermore, it plays a role in axon guidance while *Cntnap4* product belongs to the neurexin family, members of which function in the nervous system as cell adhesion molecules and receptors (Pasterkamp and Giger 2009; Yamashita et al. 2007).

Neural remodeling is a fundamental process by which the brain responds to environmental influences during stress. In PFC of stressed LA mice, we identified upregulation of three stress-responsive genes involved in myelin function: *Gpm6a*, *Mal*, and *Mog*. *Gpm6a* codes glycoprotein M6a which plays an important role in neurite outgrowth and synapse formation (Alfonso et al*.* 2005). This gene is downregulated in the hippocampus of both socially and physically stressed animals, and this effect can be reversed by antidepressant treatment (Alfonso et al. 2005). In our previous study, we found downregulation of *Gpm6a* in hippocampus of LA mice after CMS (Lisowski et al*.* 2011). GMP6A may play a role in the stress-induced hippocampal alterations that are found in psychiatric disorders (Boks et al. 2008). *Mal* encodes integral membrane protein belonging to the MAL family of proteolipids involved in myelin biogenesis and function. The product of *Mog* is a membrane protein MOG, expressed on the oligodendrocyte cell surface and the outermost surface of myelin sheaths. Downregulation of cortical *Mog* expression was found to be involved in schizophrenia and major depressive disorder (Aston et al*.* 2005; Martins-de-Souza et al. 2010). In the other hand, MOG is detectable in multiple sclerosis (MS) patients and is suspected to be involved in pathogenesis of MS (Stern and Keskin 2008). Upregulation of genes that encode structural components of myelin such as *Gpma6*, *Mal*, and *Mog* in PFC of LA mice could be an adaptive mechanism of stress response dependent on stress procedure duration or individual vulnerability.

CMS induces neuroadaptive changes that could address clinical findings in depressed patients (Frodl et al*.* 2008; Gould et al. 2007; Lucassen et al*.* 2006; Rajkowska 2000; Sanacora et al*.* 2004). Recent clinical (Uezato et al*.* 2009) and preclinical studies (Garcia-Garcia et al*.* 2009; Tordera et al. 2007) have linked decreased levels of the synaptic vesicle protein vesicular glutamate transporter 1 (VGLUT1) to depressive-like behavior. In addition, recent studies with heterozygous VGLUT1 knockout mice suggest that decreased VGLUT1 levels affects glutamate transmission and induces depressive-like behavior comorbid with anxiety and impaired recognition memory (Balschun et al*.* 2010; Tordera et al*.* 2007). On the other hand, in our study, we noticed upregulation of *VGluT1* (also *Slc17a7*) in HA CMS mice and downregulation of metabotropic glutamate receptor 5 (*Grm5*) in LA CMS mice. *Grm5* is a subtype of group I glutamate receptors, is highly expressed in limbic forebrain regions, and is thought to modulate anxiety-related processes. The blockade of *Grm5* by specific antagonist 2-methyl-6-(phenylethynyl) pyridine, reduce extracellular norepinephrine, the impact of which may contribute to their anxiolytic actions (Page et al*.* 2005). We suggest that the downregulation of *Grm5* together with disregulation of other genes included in *signal transduction* and *response to stress* clusters of LA CMS mice and upregulation of *VGluT1* could be a mechanism of adaptation to stress of stress resistant individuals. Moreover, upregulation of *VGluT1* is according to our previous study in which we have observed significant upregulation of *VGluT1* and *VGluT2* mRNA in the hippocampus of LA mice after CMS procedure (Lisowski et al*.* 2011).

In HA mice, we observed significant upregulation of arginine vasopressin transcript (*Avp*). Activation of the hypothalamic-pituitary-adrenal system by psychosocial stress is accompanied by an increase in peripheral plasma AVP levels in human study (Zimmermann et al*.* 2004). Repeated stress is known to induce an increased vasopressin (AVP) expression in paraventricular corticotrophin-releasing hormone (CRH) neurons that is supposed to enhance the ACTH-releasing capacity of these cells. Acute immobilization produced a significant increase in the average AVP and CRF mRNA levels in the medial parvocellular subdivisions of the paraventricular nucleus (PVN) (Aubry et al*.* 1999). PVN AVP mRNA levels are more sensitive to glucocorticoid negative feedback than are the levels of CRH mRNA (Makino et al. 1995). In the brain as a whole, AVP acts on wide array of neurons. AVP might also modulate emotional memory and anxiety (Koob 2008). We suggest that in CMS, a robust increase in PFC of *Avp* mRNA level is a mechanism contributing to the maintenance of a HPA response after repeated stress. Besides hypothalamus and amygdala, the PFC excitatory actions of AVP released from cortical neurons may also contribute to the behavioral stress response.

In our previous study, we found that naïve HA mice display robust upregulation of tridecapeptide neurotensin (NT) receptor transcript (*Nts2r*) in hippocampus as compared to naïve LA mice (Lisowski et al. 2012). Recently, it became evident that NT is involved in responsiveness to both pain and stressful stimuli, suggesting that this neurotransmitter may be involved in the phenomenon of non-opioid SIA (Dobner 2005). The release of endogenous NT in response to stress requires the presence of NTS2 to stimulate corticotropin-releasing factor-induced elevation of plasma corticosterone (Lafrance et al. 2010). These data highlight the significance of NTS2 as a novel target for the treatment of pain and stress-related disorders. In the current study, we observed significant upregulation of neurotensin transcript (*Nts*) in HA mice after CMS procedure. This gene encodes a common precursor for two peptides, neuromedin N and neurotensin. Neurotensin is a secreted tridecapeptide, which is widely distributed throughout the central nervous system, and may function as a neurotransmitter or a neuromodulator. It may be involved in dopamine-associated pathophysiological events. Both, *Nts* and *Drd1a* were clustered in group of genes involved in homeostatic process/regulation of biological quality in HA CMS mice. In addition to the role of NT in the regulation of nociceptive processing, there is accumulating evidence suggesting that NT is involved in hormonal, neural, and behavioral stress-related responses. NT turnover is also altered under physiological stress. Exposure to various stressors, such as immobilization or cold-water swim, induces an up-regulation of the NT precursor mRNA in several hypothalamic regions, including the medial preoptic area and the paraventricular nucleus (Ceccatelli and Orazzo 1993; Seta et al. 2001). Since there are also studies that provided the evidence for the involvement of NT in the development of neuropsychiatric disorders, we suggest that the involvement of NT in the regulation of the hypothalamo–pituitary–adrenal gland axis during chronic stress should be considered in the context of vulnerability phenotypes.

Among differentially expressed genes of *response to stress* cluster in LA CMS mice, we identified robust upregulation of calpain 2 (*Capn2*) and downregulation of corticotrophin-releasing hormone receptor 1 (*Crhr1*). Calpains are a group of calcium-dependent protease that plays a significant role in synaptic plasticity, cell motility, and neurodegeneration (Liu et al*.* 2005; Wu and Lynch 2006). Calpain-mediated spectrin degradation has been implicated in dendritic spine changes associated with LTP induction (Lynch and Baudry 1984; Vanderklish and Bahr 2000; Vanderklish et al. 2000). In addition, calpain inhibitors block LTP induction in vitro and in vivo (Denny et al*.* 1990; Staubli et al. 1988). Despite the quite well-known physiological role of m-calpain in brain, it is not clear under which conditions m-calpain could be activated (Friedrich 2004); however, based on our data, the chronic stress-dependent upregulation of *Capn2* in PFC is possible in vulnerable phenotypes.

PFC contains “hot spots” of receptors for key stress mediators such as β1-adrenoceptors (β1R), CRH receptors (CRHR1, CRHR2), mineralocorticoid, and glucocorticoid receptors (MR, GR) (Joels and Baram 2009). In LA CMS mice, we observed downregulation of *Crhr1* that encodes a G-protein-coupled receptor that binds neuropeptides of the CRH family. Actions of stress-induced CRH release are mediated primarily through binding to CRHR1 while binding to CRHR2 exert effects at long timescale and might function to shut down the stress response (Bale et al. 2000; Coste et al*.* 2000; Muller et al. 2003). Quantity of *Crhr1* mRNA in rodent PFC in contrast to *Crhr2* is high (Aguilera et al*.* 2004). Significant downregulation of *Crhr1* transcription suggests decreased CRH binding following chronic stress in PFC. It is likely that transcriptional regulatory mechanisms that permit rapid changes in *Crhr1* activity in PFC are important for adaptation of corticotroph responsiveness to continuous changes in physiological demands. In this regard, pituitary *Crhr1* mRNA levels decrease following glucocorticoid administration and recover only when circulating glucocorticoids decline below stress levels (Ochedalski et al. 1998). These findings suggest that the glucocorticoids contribute to the decrease in cortical Crhr1 mRNA during stress. The mechanism regulating PFC *Crhr1* mRNA levels during stress is likely to involve increased exposure of the cortical corticotroph to glucocorticoids, CRH and AVP.

Insulin plays a role in the structural responses of the brain to stressors. Lack of insulin causes a decrease in dentate gyrus neuron number and leads to increased remodeling of dendrites of CA3 neurons that is further accelerated by repeated restraint stress. Stress and hyperglycemia both increase oxidative stress in the brain and this is likely to contribute, over time, to impaired neural function in chronic stress and diabetes. In the present study, we found that in HA mice, genes involved in hormone secretion including insulin secretion genes were deregulated. Among them, we found upregulation of prolactin receptor (*Prlr*). Study showed that prolactin (PRL) is a neuromodulator of behavioral and neuroendocrine stress coping in the rat (Blume et al*.* 2009; Torner et al*.* 2001). Downregulation of brain prolactin receptors increased anxiety-related behavior demonstrating an anxiolytic effect of PRL acting at brain level (Torner et al. 2001). Furthermore stress-induced increase of corticotropin secretion was decreased after chronic intracerebroventricular infusion of PRL (Torner et al. 2001). Fujikawa (2004) showed that PRL levels increase in response to stress acting on the central nervous system and plays an important role in helping to protect against acute stress-induced hypocalcemia. Taken together, prolactin acting at brain level has to be considered as a novel regulator of stress response and HPA axis reactivity in PFC.

The data from the current study indicate that in addition to abnormalities related to neurons, communication in chronic stress may be altered due to functional changes in multiple components of signal transduction mechanisms. Significant numbers of differentially expressed genes in our mouse models displayed altered expression in humans with neurodegenerative diseases. Also of interest was the decreased expression of *Drd1a*, *Crhr1*, *Grm5*. Its altered activity has been associated with various human neurological disorders including schizophrenia, Alzheimer's disease and Huntington's disease. Regulation may serve as an adaptive mechanism in response to prolonged stress, and may be relevant to chronic stress-induced depression in PFC region.

## Conclusions

To summarize, transcriptional profiling revealed evidence of changes in cell systems that might contribute to structural and functional abnormalities in the prefrontal cortex in individuals with different genetic backgrounds. Further studies are necessary to confirm these findings and to determine how these changes in gene expression are switched at different time-points of chronic stress. In genetically predisposed individuals, an imbalance in control mechanisms of gene expression can introduce a bias towards stress-related brain disease after adverse experiences. New candidate genes that serve as biomarkers for the prediction of stress-vulnerable phenotypes should continue to be tested. The relevance of our findings to human stress, depression, or anxiety is yet unclear; however, our animal model for chronic stress allowed insights into molecular processes. Further assessment of alterations in gene expression in brain regions are required to determine the possible role of stressful situation during an adaptation or habituation to repeated stress. In addition, pharmacological validation should be performed to confirm that drugs active in the treatment of anxiety or depression could reversed the stress-induced gene expression alteration. It should be noted that despite the identification of different genes in each mice strain, the exposure to stress caused a similar biological effect based on GO database enrichements. The study results show that many genetic factors, not one allele, determine how an individual responds to stress and stressful situation.

## Limitations

The assessment of the possible chronic stress-related alterations in the PFC transcriptome may be relevant to the mechanisms involved in stress-induced neuropsychopathologies. Transcriptomic model reduces the biological complexity of stress response to the genetic level, whereas it should include genomic, epigenomic, and proteomic levels in the context of systems biology. Our CMS model assumes that cortical cells can modify metabolic functions related to animals' behavior. Another thing is that many metabolic or cell cycle-related genes were identified among the differentially expressed transcripts certainly points to the possibility that the observed expression patterns could be only indirectly related to distinct LA and HA mice phenotypes and may underline differences in other organs, e.g., endocrine system functions. We speculate that the selection for high and low SIA may be the basis of strain differences at the brain metabolic level. Genes are tested at one time point, often not based on a biological system, e.g., circadian genes and circadian rhythm. Thus, further studies are needed to determine how the transcriptomic profiles of particular brain structures distributing in particular time points of chronic stress to unravel the differences between adaptation and disease. Furthermore, development of mouse models where identified genes are either knocked-down or overactive and crossing them with models of, e.g., Alzheimer's disease, Parkinson's disease, multiple sclerosis or amyotrophic lateral sclerosis, to see if it can influence neurodegeneration could reveal their impact on neuropsychopathologies. Screening for epigenetics compounds as DNA methylation and non-coding RNA action that can be altered is also required. Integration of genomics and epigenomics could better explain the chronic stress-related psychopathologies.

## Electronic Supplementary Material

Below is the link to the electronic supplementary material.

ESM 1 (XLS 145 kb)

ESM 2 (XLS 55 kb)

ESM 3 (XLS 61 kb)

## References

[CR1] Aguilera G, Nikodemova M, Wynn PC, Catt KJ (2004). Corticotropin releasing hormone receptors: two decades later. Peptides.

[CR2] Airan RD, Meltzer LA, Roy M, Gong Y, Chen H, Deisseroth K (2007). High-speed imaging reveals neurophysiological links to behavior in an animal model of depression. Science.

[CR3] Alfonso J, Fernandez ME, Cooper B, Flugge G, Frasch AC (2005). The stress-regulated protein M6a is a key modulator for neurite outgrowth and filopodium/spine formation. Proc Natl Acad Sci U S A.

[CR4] Amat J, Baratta MV, Paul E, Bland ST, Watkins LR, Maier SF (2005). Medial prefrontal cortex determines how stressor controllability affects behavior and dorsal raphe nucleus. Nat Neurosci.

[CR5] Andrus BM, Blizinsky K, Vedell PT, Dennis K, Shukla PK, Schaffer DJ, Radulovic J, Churchill GA, Redei EE (2012). Gene expression patterns in the hippocampus and amygdala of endogenous depression and chronic stress models. Mol Psychiatry.

[CR6] Arnsten AF (2009). Stress signalling pathways that impair prefrontal cortex structure and function. Nat Rev Neurosci.

[CR7] Aston C, Jiang L, Sokolov BP (2005). Transcriptional profiling reveals evidence for signaling and oligodendroglial abnormalities in the temporal cortex from patients with major depressive disorder. Mol Psychiatry.

[CR8] Aubry JM, Bartanusz V, Jezova D, Belin D, Kiss JZ (1999). Single stress induces long-lasting elevations in vasopressin mRNA levels in CRF hypophysiotrophic neurones, but repeated sess is required to modify AVP immunoreactivity. J Neuroendocrinol.

[CR9] Bale TL, Contarintro A, Smith GW, Chan R, Gold LH, Sawchenko PE, Koob GF, Vale WW, Lee KF (2000). Mice deficient for corticotropin-releasing hormone receptor-2 display anxiety-like behaviour and are hypersensitive to stress. Nat Genet.

[CR10] Balschun D, Moechars D, Callaerts-Vegh Z, Vermaercke B, Van Acker N, Andries L, D’Hooge R (2010). Vesicular glutamate transporter VGLUT1 has a role in hippocampal long-term potentiation and spatial reversal learning. Cereb Cortex.

[CR11] Banasr M, Duman RS (2007). Regulation of neurogenesis and gliogenesis by stress and antidepressant treatment. CNS Neurol Disord Drug Targets.

[CR12] Bekris S, Antoniou K, Daskas S, Papadopoulou-Daifoti Z (2005). Behavioural and neurochemical effects induced by chronic mild stress applied to two different rat strains. Behav Brain Res.

[CR13] Benjamini Y, Hochberg Y (1995). Controlling the false discovery rate: a practical and powerful approach to multiple testing. J R Statist Soc B.

[CR14] Bergstrom A, Jayatissam MN, Mork A, Wiborg O (2008). Stress sensitivity and resilience in the chronic mild stress rat model of depression; an in situ hybridization study. Brain Res.

[CR15] Błaszczyk JW, Tajchert K, Łapo I, Sadowski B (2000). Acoustic startle and open-field behavior in mice bred for magnitude of swim analgesia. Physiol Behav.

[CR16] Blevins G, Macaulay R, Harder S, Fladeland D, Yamashita T, Yazaki M, Hamidi Asl K, Benson MD, Donat JR (2003). Oculoleptomeningeal amyloidosis in a large kindred with a new transthyretin variant Tyr69His. Neurol.

[CR17] Blume A, Torner L, Liu Y, Subburaju S, Aguilera G, Neumann ID (2009). Prolactin activates mitogen-activated protein kinase signaling and corticotropin releasing hormone transcription in rat hypothalamic neurons. Endocrinol.

[CR18] Boks MPM, Hoogendoorn M, Jungerius BJ, Bakker SC, Sommer IE, Sinke RJ, Ophoff RA, Kahn RS (2008). Do mood symptoms subdivide the schizophrenia phenotype? Associationof the GMP6A gene with a depression subgroup. Am J Med Genet B Neuropsychiatr Genet.

[CR19] Breier A, Davis OR, Buchanan RW (1991). Alprazolam attenuates metabolic stress-induced neuroendocrine and behavioral effects in humans. Psychopharmacol.

[CR20] Brown SM, Henning S, Wellman CL (2005). Mild, short-term stress alters dendritic morphology in rat medial prefrontal cortex. Cereb Cortex.

[CR21] Butler RK, Finn DP (2009). Stress-induced analgesia. Prog Neurobiol.

[CR22] Cahoy JD, Emery B, Kaushal A, Foo LC, Zamanian JL, Christopherson KS, Xing Y, Lubischer JL, Krieg PA, Krupenko SA, Thompson WJ, Barres BA (2008). A transcriptome database for astrocytes, neurons, and oligodendrocytes: a new resource for understanding brain development and function. J Neurosci.

[CR23] Ceccatelli S, Orazzo C (1993). Effect of different types of stressors on peptide messenger ribonucleic acids in the hypothalamic paraventricular nucleus. Acta Endocrinol.

[CR24] Coste SC, Kesterson RA, Heldwein KA, Stevens SL, Heard AD, Hollis JH, Murray SE, Hill JK, Pantely GA, Hohimer AR, Hatton DC, Phillips TJ, Finn DA, Low MJ, Rittenberg MB, Stenzel P, Stenzel-Poore MP (2000). Abnormal adaptations to stress and impaired cardiovascular function in mice lacking corticotropin-releasing hormone receptor-2. Nat Genet.

[CR25] Cummings JL (1992). Depression and Parkinson’s disease: a review. Am J Psychiatry.

[CR26] Czeh B, Perez-Cruz C, Fuchs E, Flugge G (2008). Chronic stress-induced cellular changes in the medial prefrontal cortex and their potential clinical implications: Does hemisphere location matter?. Behav Brain Res.

[CR27] D’Aquila PS, Brain P, Willner P (1994). Effects of chronic mild stress on performance in behavioural tests relevant to anxiety and depression. Physiol Behav.

[CR28] Denny JB, Polan-Curtain J, Ghuman A, Wayner MJ, Armstrong DL (1990). Calpain inhibitors block long-term potentiation. Brain Res.

[CR29] Deutch AY (1993). Prefrontal cortical dopamine systems and the elaboration of functional corticostriatal circuits: implications for schizophrenia and Parkinson's disease. J Neural Transm Gen Sect.

[CR30] Dobner PR (2005). Multitasking with neurotensin in the central nervous system. Cell Mol Life Sci.

[CR31] Dohrenwend BP (1994). Psychology, psychologists, and psychiatric epidemiology. Acta Psych Scan.

[CR32] Dolan RJ, Bench CJ, Brown RG, Scott LC, Frackowiak RS (1994). Neuropsychological dysfunction in depression: the relationship to regional cerebral blood flow. Psych Med.

[CR33] Drevets WC (2000). Neuroimaging studies of mood disorders. Biol Psychiatry.

[CR34] Drevets WC, Price JL, Simpson JR, Todd RD, Reich T, Vannier M, Raichle ME (1997). Subgenual prefrontal cortex abnormalities in mood disorders. Nature.

[CR35] Elizalde N, Gil-Beam FJ, Ramirez MJ, Aisa B, Lasheras B, Del Rio J, Tordera RM (2008). Long-lasting behavioral effects and recognition memory deficit induced by chronic mild stress in mice: effect of antidepressant treatment. Psychopharmacol.

[CR36] Fibiger HC (1995). Neurobiology of depression: focus on dopamine. Adv Bioch Psychopharmacol.

[CR37] Friedrich P (2004). The intriguing Ca2+ requirement of calpain activation. Biochem Biophys Res Commun.

[CR38] Frodl TS, Koutsouleris N, Bottlender R, Born C, Jager M, Scupin I, Reiser M, Moller HJ, Meisenzahl EM (2008). Depression-related variation in brain morphology over 3 years: effects of stress?. Arch Gen Psychiatry.

[CR39] Fujikawa T, Soya H, Tamashiro KL, Sakai RR, McEwen BS, Nakai N, Ogata M, Suzuki I, Nakashima K (2004). Prolactin prevents acute stress-induced hypocalcemia and ulcerogenesis by acting in the brain of rat. Endocrinol.

[CR40] Garcia-Garcia AL, Elizalde N, Matrov D, Harro J, Wojcik SM, Venzala E, Ramirez MJ, Del Rio J, Tordera RM (2009). Increased vulnerability to depressive-like behavior of mice with decreased expression of VGLUT1. Biol Psychiatry.

[CR41] Garzuly F, Vidal R, Wisniewski T, Brittig F, Budka H (1996). Familial meningocerebrovascular amyloidosis, Hungarian type, with mutant transthyretin (TTR Asp18Gly). Neurol.

[CR42] Gould NF, Holmes MK, Fantie BD, Luckenbaugh DA, Pine DS, Gould TD, Burgess N, Manji HK, Zarate CA (2007). Performance on a virtual reality spatial memory navigation task in depressed patients. Am J Psychiatry.

[CR43] Gronli J, Fiske E, Murison R, Bjorvatn B, Sorensen E, Ursin R, Portas CM (2007). Extracellular levels of serotonin and GABA in the hippocampus after chronic mild stress in rats. A microdialysis study in an animal model of depression. Behav Brain Res.

[CR44] Hamon M (2006) Science vision. University of Maastricht

[CR45] Han JS, Bizon JL, Chun HJ, Maus CE, Gallagher M (2002). Decreased glucocorticoid receptor mRNA and dysfunction of HPA axis in rats after removal of the cholinergic innervation to hippocampus. Eur J Neurosci.

[CR46] Herman JP, Figueiredo H, Mueller NK, Ulrich-Lai Y, Ostrander MM, Choi DC, Cullinan WE (2003). Central mechanisms of stress integration: hierarchical circuitry controlling hypothalamo-pituitary-adrenocortical responsiveness. Front Neuroendocrinol.

[CR47] Hugues S, Deschaux O, Garcia R (2004). Postextinction infusion of a mitogen-activated protein kinase inhibitor into the medial prefrontal cortex impairs memory of the extinction of conditioned fear. Learn Mem.

[CR48] Izquierdo A, Wellman CL, Holmes A (2006). Brief uncontrollable stress causes dendritic retraction in infralimbic cortex and resistance to fear extinction in mice. J Neurosci.

[CR49] Jayatissa MN, Bisgaard CE, West MJ, Wiborg O (2008). The number of granule cells in rat hippocampus is reduced after chronic mild stress and re-established after chronic escitalopram treatment. Neuropharmacol.

[CR50] Joels M, Baram TZ (2009). The neuro-symphony of stress. Nat Rev Neurosci.

[CR51] Joels M, Karst H, Krugers HJ, Lucassen PJ (2007). Chronic stress: implications for neuronal morphology, function and neurogenesis. Front Neuroendocrinol.

[CR52] Jones LA, Skare JC, Cohen AS, Harding JA, Milunsky A, Skinner M (1992). Familial amyloidotic polyneuropathy: a new transthyretin position 30 mutation (alanine for valine) in a family of German descent. Clin Genet.

[CR53] Juszczak GR, Sliwa AT, Wolak P, Tymosiak-Zielinska A, Lisowski P, Swiergiel AH (2006). The usage of video analysis system for detection of immobility in the tail suspension test in mice. Pharmacol Biochem Behav.

[CR54] Juszczak GR, Błaszczyk J, Sadowski B, Sliwa AT, Wolak P, Tymosiak-Zielinska A, Lisowski P, Swiergiel AH (2008). Lipopolysaccharide does not affect acoustic startle reflex in mice. Brain Behav Immun.

[CR55] Juszczak GR, Lisowski P, Sliwa AT, Swiergiel AH (2008). Computer assisted video analysis of swimming performance in a forced swim test: simultaneous assessment of duration of immobility and swimming style in mice selected for high and low swim-stress induced analgesia. Physiol Behav.

[CR56] Karssen AM, Her S, Li JZ, Patel PD, Meng F, Bunney WE, Jones EG, Watson SJ, Akil H, Myers RM, Schatzberg AF, Lyons DM (2007). Stress-induced changes in primate prefrontal profiles of gene expression. Mol Psychiatry.

[CR57] Koob GF (2008). A role for brain stress systems in addiction. Neuron.

[CR58] Lafrance M, Roussy G, Belleville K, Maeno H, Beaudet N, Wada K, Sarret P (2010). Involvement of NTS2 receptors in stress-induced analgesia. Neurosci.

[CR59] Landers KA, McKinnon BD, Li H, Subramaniam VN, Mortimer RH, Richard K (2009). Carrer-mediated thyroid hormone transport into placenta by placental transthyretin. J Clin Endocrinol Metab.

[CR60] Li CS, Sinha R (2008). Inhibitory control and emotional stress regulation: neuroimaging evidence for frontal-limbic dysfunction in psycho-stimulant addiction. Neurosci Biobehav Rev.

[CR61] Lisowski P, Robakowska-Hyzorek D, Blitek A, Kaczmarek MM, Gajewska A, Kochman H, Zwierzchowski L, Ziecik AJ, Kochman K (2008). Development of real-time PCR assays in the study of gonadotropin subunits, follistatin and prolactin genes expression in the porcine anterior pituitary during the preovulatory period. Neuro Endocrinol Lett.

[CR62] Lisowski P, Pierzchała M, Gościk J, Pareek CS, Zwierzchowski L (2008). Evaluation of reference genes for studies of gene expression in the bovine liver, kidney, pituitary, and thyroid. J Appl Genet.

[CR63] Lisowski P, Juszczak GR, Goscik J, Wieczorek M, Zwierzchowski L, Swiergiel AH (2011). Effect of chronic mild stress on hippocampal transcriptome in mice selected for high and low stress-induced analgesia and displaying different emotional behaviors. Eur Neuropsychopharmacol.

[CR64] Lisowski P, Stankiewicz AM, Goscik J, Wieczorek M, Zwierzchowski L, Swiergiel AH (2012). Selection for stress responsiveness affects the mouse hippocampal transcriptome. J Mol Neurosci.

[CR65] Liu F, Grundke-Iqbal I, Iqbal K, Oda Y, Tomizawa K, Gong CX (2005). Truncation and activation of calcineurin A by calpain I in Alzheimer disease brain. J Biol Chem.

[CR66] Lucassen PJ, Heine VM, Muller MB, van der Beek EM, Wiegant VM, De Kloet ER, Joels M, Fuchs E, Swaab DF, Czeh B (2006). Stress, depression and hippocampal apoptosis. CNS & Neurol Disord Drug Targets.

[CR67] Lynch G, Baudry M (1984). The biochemistry of memory: a new and specific hypothesis. Science.

[CR68] Makino S, Smith MA, Gold PW (1995). Increased expression of corticotropin-releasing hormone and vasopressin messenger ribonucleic acid (mRNA) in the hypothalamic paraventricular nucleus during repeated stress: association with reduction in glucocorticoid receptor mRNA levels. Endocrinol.

[CR69] Malmberg AB, Chen C, Tonegawa S, Basbaum AI (1997). Preserved acute pain and reduced neuropathic pain in mice lacking PKCgamma. Science.

[CR70] Martins-de-Souza D, Harris LW, Guest PC, Turck CW, Bahn S (2010). The role of proteomics in depression research. Eur Arch Psychiatry Clin Neurosci.

[CR71] Mazure CM, Kincare P, Schaffer CE (1995). DSM-III-R Axis IV: clinician reliability and comparability to patients' reports of stressor severity. Psychiatry.

[CR72] McEwen BS (2004). Protection and damage from acute and chronic stress: allostasis and allostatic overload and relevance to the pathophysiology of psychiatric disorders. Ann N Y Acad Sci.

[CR73] McEwen BS (2006). Protective and damaging effects of stress mediators: central role of the brain. Dialogues Clin Neurosci.

[CR74] Meeus M, Nijs J, Van de Wauwer N, Toeback L, Truijen S (2008). Diffuse noxious inhibitory control is delayed in chronic fatigue syndrome: an experimental study. Pain.

[CR75] Miner LH, Jedema HP, Moore FW, Blakely RD, Grace AA, Sesack SR (2006). Chronic stress increases the plasmalemmal distribution of the norepinephrine transporter and the coexpression of tyrosine hydroxylase in norepinephrine axons in the prefrontal cortex. J Neurosci.

[CR76] Mizoguchi K, Yuzurihara M, Ishige A, Sasaki H, Chui DH, Tabira T (2000). Chronic stress induces impairment of spatial working memory because of prefrontal dopaminergic dysfunction. J Neurosci.

[CR77] Mizoguchi K, Yuzurihara M, Ishige A, Sasaki H, Tabira T (2002). Chronic stress impairs rotarod performance in rats: implications for depressive state. Pharmacol Biochem Behav.

[CR78] Muller MB, Zimmermann S, Sillaber I, Hagemeyer TP, Deussing JM, Timpl P, Kormann MS, Droste SK, Kuhn R, Reul JM, Holsboer F, Wurst W (2003). Limbic corticotropin-releasing hormone receptor 1 mediates anxiety-related behavior and hormonal adaptation to stress. Nat Neurosci.

[CR79] Murakami T, Yi S, Yamamoto K, Maruyama S, Araki S (1992). Familial amyloidotic polyneuropathy: report of patients heterozygous for the transthyretin Gly42 gene. Annal Neurol.

[CR80] Murakami T, Tachibana S, Endo Y, Kawai R, Hara M, Tanase S, Ando M (1994). Familial Carpal-Tunnel Syndrome Due to Amyloidogenic Transthyretin His-114 Variant. Neurol.

[CR81] Nishith P, Griffin MG, Poth TL (2002). Stress-induced analgesia: prediction of posttraumatic stress symptoms in battered versus nonbattered women. Biol Psychiatry.

[CR82] Ochedalski T, Rabadan-Diehl C, Aguilera G (1998). Interaction between glucocorticoids and corticotropin releasing hormone (CRH) in the regulation of the pituitary CRH receptor in vivo in the rat. J Neuroendocrinol.

[CR83] Page AJ, Young RL, Martin CM, Umaerus M, O’Donnell TA, Cooper NJ, Coldwell JR, Hulander M, Mattsson JP, Lehmann A, Blackshaw LA (2005). Metabotropic glutamate receptors inhibit mechanosensitivity in vagal sensory neurons. Gastroenterol.

[CR84] Panocka I, Marek P, Sadowski B (1986). Differentiation of neurochemical basis of stress-induced analgesia in mice by selective breeding. Brain Res.

[CR85] Panocka I, Marek P, Sadowski B (1986). Inheritance of stress-induced analgesia in mice. Selective breeding study. Brain Res.

[CR86] Panocka I, Massi M, Łapo I, Swiderski T, Kowalczyk M, Sadowski B (2001). Antidepressant-type effect of the NK3 tachykinin receptor agonist aminosenktide in mouse lines differing in endogenous opioid system activity. Peptides.

[CR87] Pascucci T, Ventura R, Latagliata EC, Cabib S, Puglisi-Allegra S (2007). The medial prefrontal cortex determines the accumbens dopamine response to stress through the opposing influences of norepinephrine and dopamine. Cereb Cortex.

[CR88] Pasterkamp RJ, Giger RJ (2009). Semaphorin function in neural plasticity and disease. Curr Opin Neurobiol.

[CR89] Pfaffl MW (2001). A new mathematical model for relative quantification in real-time RT-PCR. Nucleic Acids Res.

[CR90] Pielsticker A, Haag G, Zaudig M, Lautenbacher S (2005). Impairment of pain inhibition in chronic tension-type headache. Pain.

[CR91] Pirot S, Jay TM, Glowinski J, Thierry AM (1994). Anatomical and electrophysiological evidence for an excitatory amino acid pathway from the thalamic mediodorsal nucleus to the prefrontal cortex in the rat. Eur J Neurosci.

[CR92] Rajkowska G (2000). Postmortem studies in mood disorders indicate altered numbers of neurons and glial cells. Biol Psychiatry.

[CR93] Ramos BP, Arnsten AF (2007). Adrenergic pharmacology and cognition: focus on the prefrontal cortex. Pharmacol Ther.

[CR94] Robbins TW (1996). Dissociating executive functions of the prefrontal cortex. Philos Trans R Soc Lond B Biol Sci.

[CR95] Sanacora G, Gueorguieva R, Epperson CN, Wu YT, Appel M, Rothman DL, Krystal JH, Mason GF (2004). Subtype-specific alterations of gamma-aminobutyric acid and glutamate in patients with major depression. Arch Gen Psychiatry.

[CR96] Saraiva MJ, Almeida Mdo R, Sherman W, Gawinowicz M, Costa P, Costa PP, Goodman DS (1992). A new transthyretin mutation associated with amyloid cardiomyopathy. Am J Hum Genet.

[CR97] Seta KA, Jansen HT, Kreitel KD, Lehman M, Behbehani MM (2001). Cold water swim stress increases the expression of neurotensin mRNA in the lateral hypothalamus and medial preoptic regions of the rat brain. Brain Res Mol Brain Res.

[CR98] Sousa JC, Grandela C, Fernandez-Ruiz J, de Miguel R, de Sousa L, Magalhaes AI, Saraiva MJ, Sousa N, Palha JA (2004). Transthyretin is involved in depression-like behaviour and exploratory activity. J Neurochem.

[CR99] Staubli U, Larson J, Thibault O, Baudry M, Lynch G (1988). Chronic administration of a thiol-proteinase inhibitor blocks long-term potentiation of synaptic responses. Brain Res.

[CR100] Staud R, Robinson ME, Vierck CJ, Cannon RC, Mauderli AP, Price DD (2003). Ratings of experimental pain and pain-related negative affect predict clinical pain in patients with fibromyalgia syndrome. Pain.

[CR101] Stern JN, Keskin DB (2008). Strategies for the identification of loci responsible for the pathogenesis of multiple sclerosis. Cell Mol Biol Lett.

[CR102] Teixeira PF, Cerca F, Santos SD, Saraiva MJ (2006). Endoplasmic reticulum stress associated with extracellular aggregates. Evidence from transthyretin deposition in familial amyloid polyneuropathy. J Biol Chem.

[CR103] Tordera RM, Totterdell S, Wojcik SM, Brose N, Elizalde N, Lasheras B, Del Rio J (2007). Enhanced anxiety, depressive-like behaviour and impaired recognition memory in mice with reduced expression of the vesicular glutamate transporter 1 (VGLUT1). Eur J Neurosci.

[CR104] Tordera RM, Garcia-Garcia AL, Elizalde N, Segura V, Aso E, Venzala E, Ramirez MJ, Del Rio J (2011). Chronic stress and impaired glutamate function elicit a depressive-like phenotype and common changes in gene expression in the mouse frontal cortex. Eur Neuropsychopharmacol.

[CR105] Torner L, Toschi N, Pohlinger A, Landgraf R, Neumann ID (2001). Anxiolytic and anti-stress effects of brain prolactin: improved efficacy of antisense targeting of the prolactin receptor by molecular modeling. J Neurosci.

[CR106] Uezato A, Meador-Woodruff JH, McCullumsmith RE (2009). Vesicular glutamate transporter mRNA expression in the medial temporal lobe in major depressive disorder, bipolar disorder, and schizophrenia. Bipolar Disord.

[CR107] Vanderklish PW, Bahr BA (2000). The pathogenic activation of calpain: a marker and mediator of cellular toxicity and disease states. Int J Exp Pathol.

[CR108] Vanderklish PW, Krushel LA, Holst BH, Gally JA, Crossin KL, Edelman GM (2000). Marking synaptic activity in dendritic spines with a calpain substrate exhibiting fluorescence resonance energy transfer. Proc Natl Acad Sci U S A.

[CR109] Vertes RP (2006). Interactions among the medial prefrontal cortex, hippocampus and midline thalamus in emotional and cognitive processing in the rat. Neuroscience.

[CR110] Wang YL, Barbacioru C, Hyland F, Xiao WM, Hunkapiller KL, Blake J, Chan F, Gonzalez C, Zhang L, Samaha RR (2006). Large scale real-time PCR validation on gene expression measurements from two commercial long-oligonucleotide microarrays. Bmc Genomics.

[CR111] Warner-Schmidt JL, Duman RS (2006). Hippocampal neurogenesis: opposing effects of stress and antidepressant treatment. Hippocampus.

[CR112] Willner P, Towell A, Sampson D, Sophokleous S, Muscat R (1987). Reduction of sucrose preference by chronic unpredictable mild stress, and its restoration by a tricyclic antidepressant. Psychopharmacol.

[CR113] Wood GE, Norris EH, Waters E, Stoldt JT, McEwen BS (2008). Chronic immobilization stress alters aspects of emotionality and associative learning in the rat. Behav Neurosci.

[CR114] Wu HY, Lynch DR (2006). Calpain and synaptic function. Mol Neurobiol.

[CR115] Yamashita N, Morita A, Ushida Y, Nakamura F, Usui H, Ohshima T, Taniguchi M, Honnorat J, Thomasset N, Takei K (2007). Regulation of spine development by semaphorin3A through cyclin-dependent kinase 5 phosphorylation of collapsin response mediator protein 1. J Neurosci.

[CR116] Zimmermann U, Spring K, Wittchen HU, Himmerich H, Landgraf R, Uhr M, Holsboer F (2004). Arginine vasopressin and adrenocorticotropin secretion in response to psychosocial stress is attenuated by ethanol in sons of alcohol-dependent fathers. J Psychiatr Res.

